# Geometrical analysis of motion schemes on fencing experts from competition videos

**DOI:** 10.1371/journal.pone.0261888

**Published:** 2021-12-30

**Authors:** Christophe Magnani, Elise Defrasne Ait-Said

**Affiliations:** Centre Borelli UMR 9010, Université de Paris, CNRS, Paris, France; Universiteit Hasselt Faculteit Wetenschappen, BELGIUM

## Abstract

Geometrical fencing is a scientific approach to fencing pioneered by Camillo Agrippa in the XVIth century which consists of characterizing the geometrical structure of fencing movements. Many geometrical spaces are involved in a duel, which evolve over time according to the skills of the fencers and the game rules. In this article, the concept of motion scheme is introduced as a flexible geometrical structure to represent fencing spaces evolving over time. The method is applied to the video of a duel of the Olympic games 2016. Five main results are presented. First, decisive actions of the duel are deduced from the distance between fencers. Second, footwork is reconstructed from horizontal movements of the feet. Third, a kinematic model is developed and compared with data in the literature. Fourth, the lunge attack is characterized and compared with data in the literature. Fifth, the role of the free hand is studied in the case of protective and balancing gestures. These findings provide rich information on the geometrical structure of fencing movements as well as on the tactical-strategic choices made by the fencers in real competition conditions. Finally, four applications illustrate the scientific value of motion schemes in fencing and other sports.

## Introduction

In 1553, the Italian fencing theorist Camillo Agrippa published the popular book *Trattato di scientia d’arme* [1], which presents the foundations of fencing that are still effective today. He was influenced by Euclidean geometry and Aristotelian physics, and as such is considered the pioneer of the scientific approach called geometrical fencing. A summary of his work can be found in Mondschein [2].

According to this approach, the space of the human body is described by geometrical entities (points, lines, …) and time is the number of motion in the Aristotle’s perspective. At that time, without advanced technology instruments, space and time were measured in relative terms using proportions instead of absolute measurements.

Agrippa explains that the thrust being a straight-line attack, it covers the shortest distance [3]. He recommends a guard position where the arm is in front of the body and the point threatens the enemy [2]. Then, he gives a geometrical argument for the lunge with a diagram, showing how the arm extension and the knee flexion allow to reach the target at distance [2]. His approach may have been inspired by mathematical methods of his time, such as the ballistic parabolas of Tartaglia. He may also have been influenced by the circular design of Cesariano’s Vitruvius, according to which the human body is symmetrical on a metric grid [4]. As such, the spherical human body is free in its movements to perform all fencing actions. Agrippa also illustrates the rays of the eyes looking at the opponent, which evokes the medieval optical theory used by Alberti to describe perspective [5].

In his day, Agrippa did not have the mathematical tools necessary for a complete quantitative analysis of fencing. Nevertheless, he established a geometrical methodology based on principles which remain fundamental today.

The purpose of this article is to revisit Agrippa’s geometrical fencing with modern quantitative methods applied to videos of duels filmed in real field conditions. The concept of motion scheme is introduced to represent fencing duels by a modular and dynamic structure organized as a directed graph of deformable spaces evolving along their time flows.

The original results obtained with this approach are illustrated on a duel of the Olympic games 2016 between two international experts, Steffen and Grumier. They demonstrate that poor quality videos taken on YouTube can contain rich information consistent with sophisticated measurements published in the literature.

The topics covered in this article are key concepts considered by fencing masters and theorists, namely duel profile, footwork, kinematic model, lunge and free hand. These are complemented by applications that illustrate the scientific value of motion schemes, namely comparison between lunge and fleche, performance of athletes in a counter-attack, new insight on dominance in duels and transferability to other sports.

## Materials and methods

### Video analysis

Elite fencing athletes must fully deploy their physical and psychological capacities during real competitions, which are difficult to reproduce in laboratory. Scientific observation of international competitions directly on the ground is expensive and installation of equipment is not always possible according to the local legislation. An affordable solution is to analyze videos of official competitions, as they are widely available on YouTube and other websites. This method has the advantage of offering abundant data with a large number of criteria (gender, ranking, country, …). The videos have been manually selected according to three quality criteria:

- Movements must be judged correct and representative by fencing experts
- Camera must have sufficient resolution and speed (≥ 25 images per second)
- View angle must encompass entire movements and avoid sliding effects

**Fig 1:**
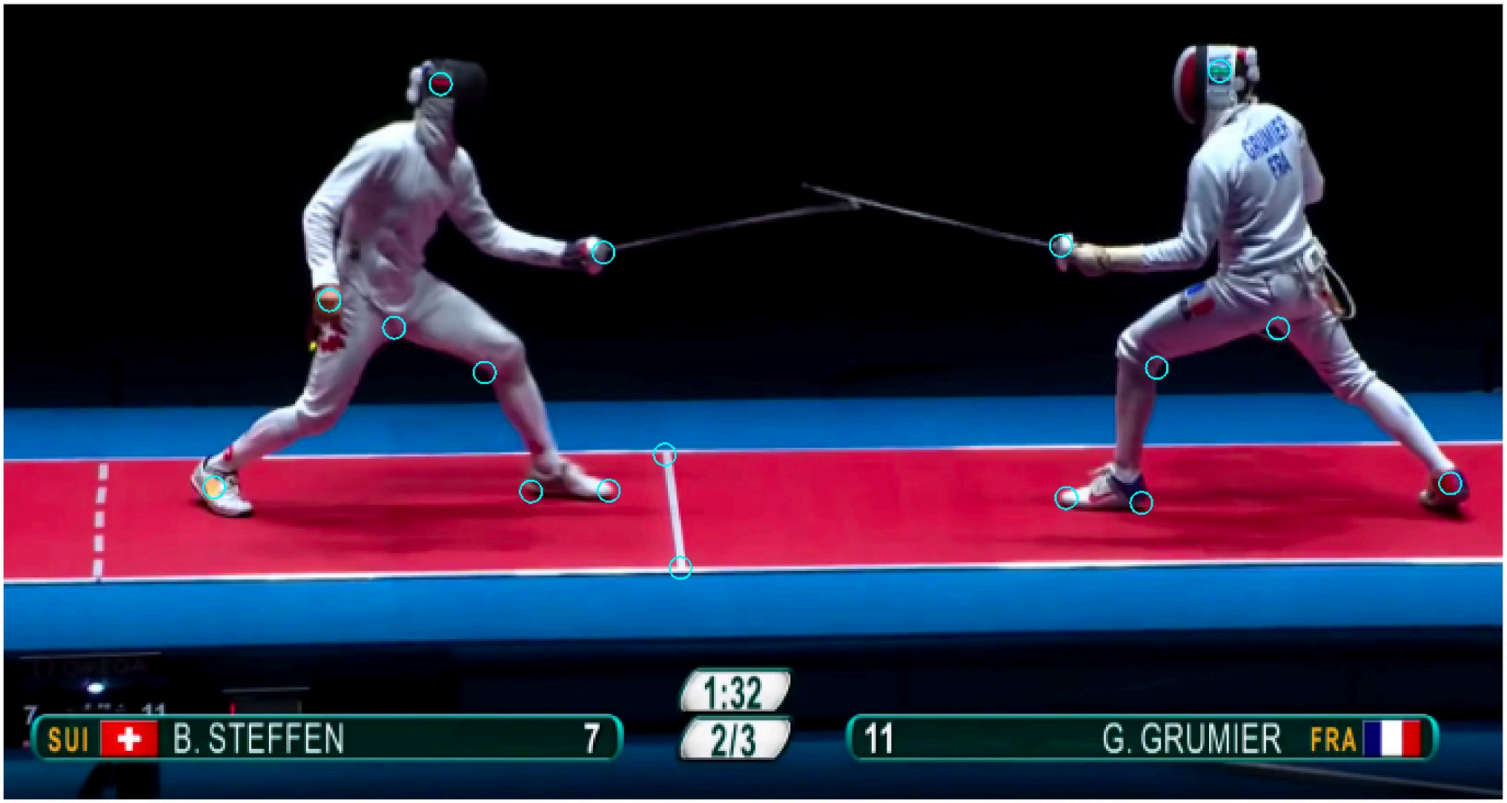
A duel at the Olympic games 2016. Left: Steffen (Swiss). Right: Grumier (France). The blue markers represent the points of interest captured by video tracking. Source of the original video: [6].

Fig 1 shows the video selected for this article, which is an épée duel at Olympic games 2016 [6] between Gauthier Grumier (France) and Bennie Steffen (Swiss). The camera quality is 1920x1080 pixels at 25 images per second (sampling time 0.04s). The sequence has 92 frames (duration 3.64s). The sequence was chosen for a direct thrust in which the hit occurs at frame #75 when Steffen’s red lamp becomes illuminated. The view angle captures the scene in the sagittal plane (xy) of the fencers. The x coordinate encodes the horizontal position along the piste. The y coordinate encodes the vertical position, however it can be slightly altered when the fencers slide slightly out of the sagittal plane.

The mathematical analysis was done using Matlab. The video editing was done using Shotcut software. The video tracking was done with custom algorithms using Python 3 and OpenCV 4. Trajectories in the plane (xy) retrace the time evolution of the points of interest defined on the scene (blue markers in Fig 1). Trajectories are distorted when the camera is zoomed, rotated or translated. Thus, the video sequence was carefully chosen to exclude such instabilities. Nevertheless, the camera moves horizontally to crop the fencers during the direct thrust, so a calibration was necessary by following two fixed points on the piste.

At each instant, geometrical data are encoded in a landmark space (Bauer et al. [7])

$$L^{n}=\{(q_{1},\ldots ,q_{n})|q_{k}\in R^{2}\}$$

A landmark $q\in L^{n}$ is a labeled collection of n points in $R^{2}$, each one tracking a specific point of interest on the video. The following configuration was chosen for its relevance to fencing experts and image quality constraints:

- 8 markers on Steffen (head, sword hand, free hand, crotch, front knee, back foot, top of front foot, heel of front foot)
- 7 markers on Grumier (head, sword hand, crotch, front knee, back foot, top of front foot, heel of front foot)
- 2 markers on the piste (fixed points)

**Fig 2:**
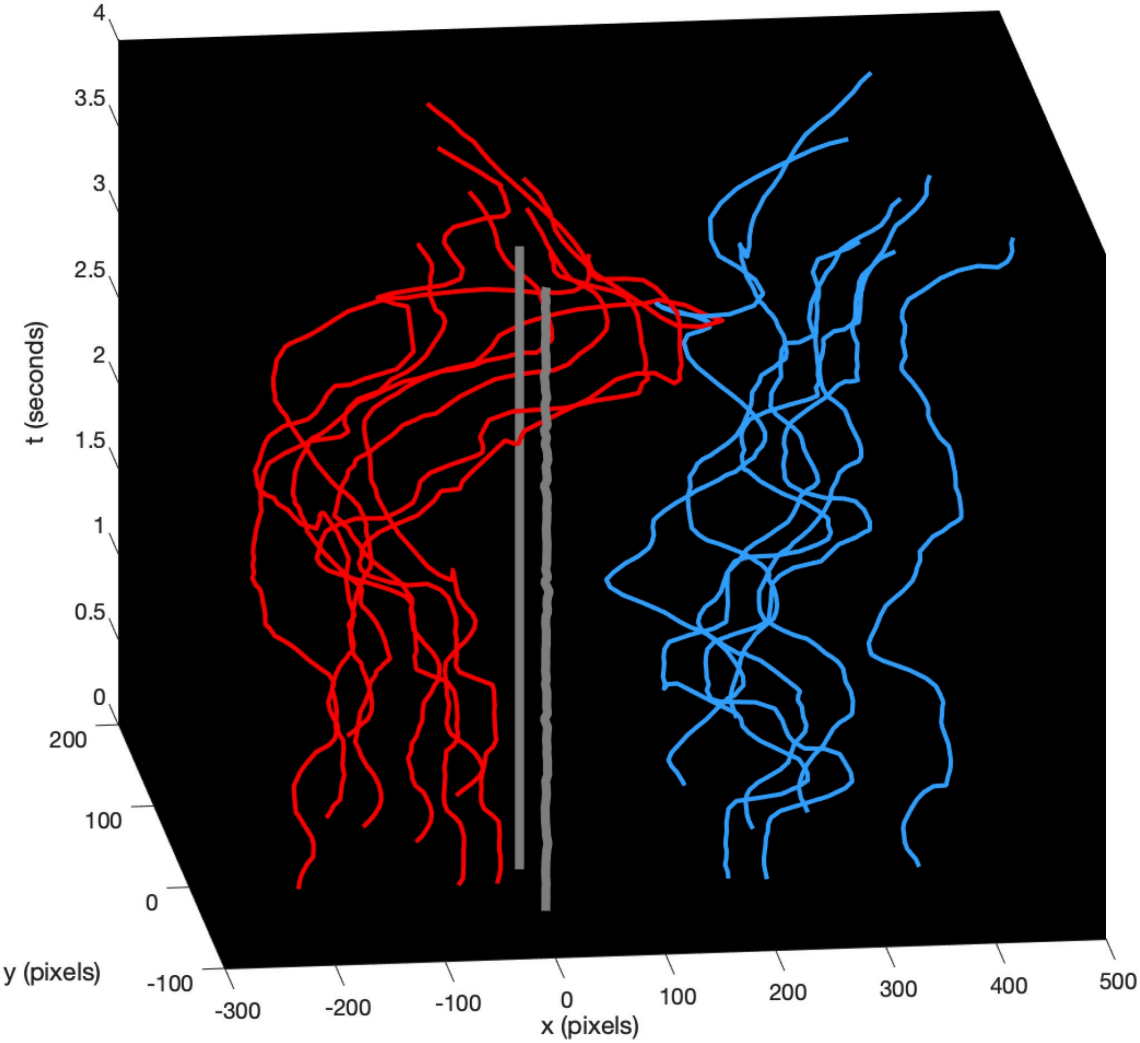
Landmark trajectories as 3D worldlines. Each worldline represents the trajectory of a marker for Steffen (red), Grumier (blue) and the piste (gray). The front view (xt) reflects fencing actions dominated by the horizontal coordinate. A net movement of Steffen towards Grumier characterizes the direct thrust.

A time-dependent landmark *q*(*t*) for *t*_*i*_ ≤ *t* ≤ *t*_*f*_ describes trajectories of points of interest between an initial configuration *q*(*t*_*i*_) and a final configuration *q*(*t*_*f*_). Each trajectory *q*_*k*_(*t*) can be represented by a 2D curve in the two-dimensional space (xy), or by a 3D worldline with time spatialized in the three-dimensional space (xyt). The 3D representation is more expressive than the 2D because it avoids self-intersections and allows comparisons of worldlines at precise time slices. Fig 2 shows the worldlines of Steffen (red), Grumier (blue) and the piste (gray). The front view (xt) in 3D perspective reflects fencing actions which are dominated by the horizontal coordinate along the piste. The worldlines of the piste are straight because they represent fixed points in the environment. The figure reveals a net movement of Steffen towards Grumier during the direct thrust.

### Motion schemes

Fencing requires precise execution of movements in space to touch the opponent without being hit oneself. Space embodies the movements made to reach a target, according to Poincaré [8]. Thus, the space of fencing is not unitary and static but rather modular and dynamic. Fencing movements determine units of space in which they take place. Each duel is organized in many geometrical spaces that are subtly intertwined. These spaces evolve over time according to the skills of the fencers and the game rules. The scientific challenge is to investigate the structure of these spaces and their role in the execution of a duel at a fundamental level.

This leads to introduce the notion of motion space as a dynamic region of interest, such as a skeletal posture, a gesture or an interaction between fencers. Such a region depends on the relationships between points of interest in the landmark controlled by voluntary movements and biomechanical constraints. Then, the concept of motion scheme will describe the time evolution of motion spaces during a duel.

We define a **motion space** *X* as a two-dimensional CW-complex (Hatcher [9]) constructed as a nested sequence of skeleta *X*_0_ ⊂ *X*_1_ ⊂ *X*_2_ such that:

- *X*_0_ contains points of interest in a landmark
- *X*_1_ contains line segments with end points glued in *X*_0_
- *X*_2_ contains polygons with edges glued in *X*_1_

In this way, motion spaces are based on relationships between points of interest defined by fencing experts. Analysis of m-ary relationships was more specifically investigated by Qu et al. [10] in graph drawing.

A time-dependent motion space *X*(*t*) for *t*_*i*_ ≤ *t* ≤ *t*_*f*_ deforms according to the trajectories of the underlying time-dependent landmark. Such a transformation determines a transition from an initial state to a final state

$$\begin{array}{c} t_{i} \end{array}\overset{X(t)}{\overset{\;}{\to }}\begin{array}{c} t_{f} \end{array}$$

We define a **motion scheme** as a transition system (*V*, *A*, Λ, *i*, *f*, *τ*, *μ*) realized by motion spaces as follows:

$$\Lambda \overset{\mu }{⟵}A\underset{i}{\overset{f}{⇉}}V\overset{\tau }{⟶}R$$

- *V* is a finite set of states (or vertices)
- *A* is a finite set of transitions (or arrows)
- Λ is a finite set of time-dependent motion spaces
- *i*: *A* → *V* associates an initial state to each transition
- *f*: *A* → *V* associates a final state to each transition
-
τ:V→R associates a timestamp to each state
- *μ*: *A* → Λ associates a time-dependent motion space to each transition
- If *a* ∈ *A* then *τ* ∘ *i*(*a*) ≤ *τ* ∘ *f*(*a*) (causality)
- If *a* ∈ *A* then *μ*(*a*)(*t*) is defined for *τ* ∘ *i*(*a*) ≤ *t* ≤ *τ* ∘ *f*(*a*) (consistency)

Thus, a motion scheme is given by a directed graph in which each arrow describes the movement of a dynamic region of interest (motion space). Each state *v* ∈ *V* is associated with a timestamp *τ*(*v*). Each transition *a* ∈ *A* is associated with a time-dependent motion space *μ*(*a*)(*t*) evolving from *μ*(*a*)(*τ* ∘ *i*(*a*)) to *μ*(*a*)(*τ* ∘ *f*(*a*)). Movements in a motion scheme are ephemeral according to their time intervals. Multiple arrows are allowed between two vertices to represent simultaneous movements.

## Results

### Duel profile

**Fig 3:**
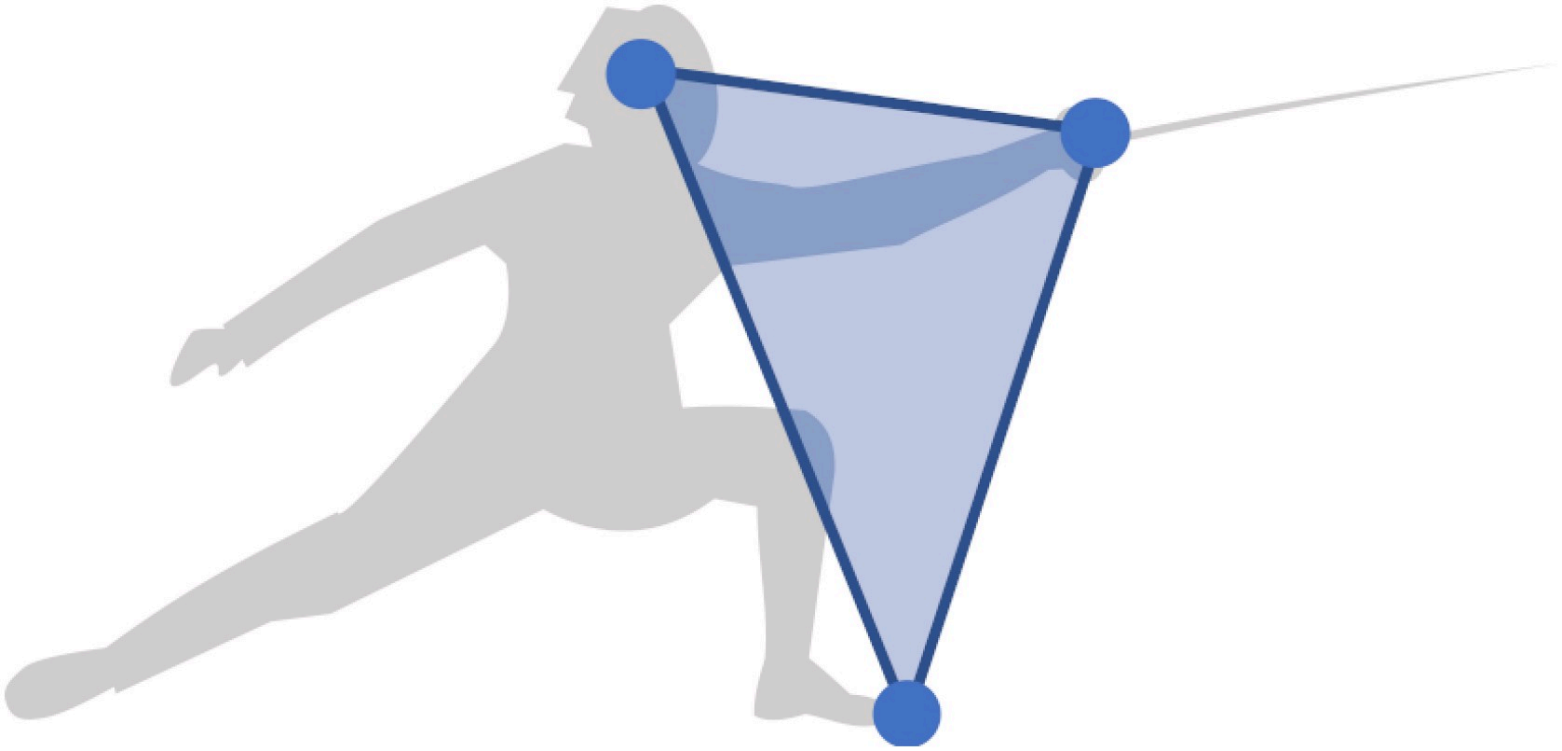
Frontal space. It is a strategic region defined between the head, the sword hand and the top of the front foot.

**Fig 4:**
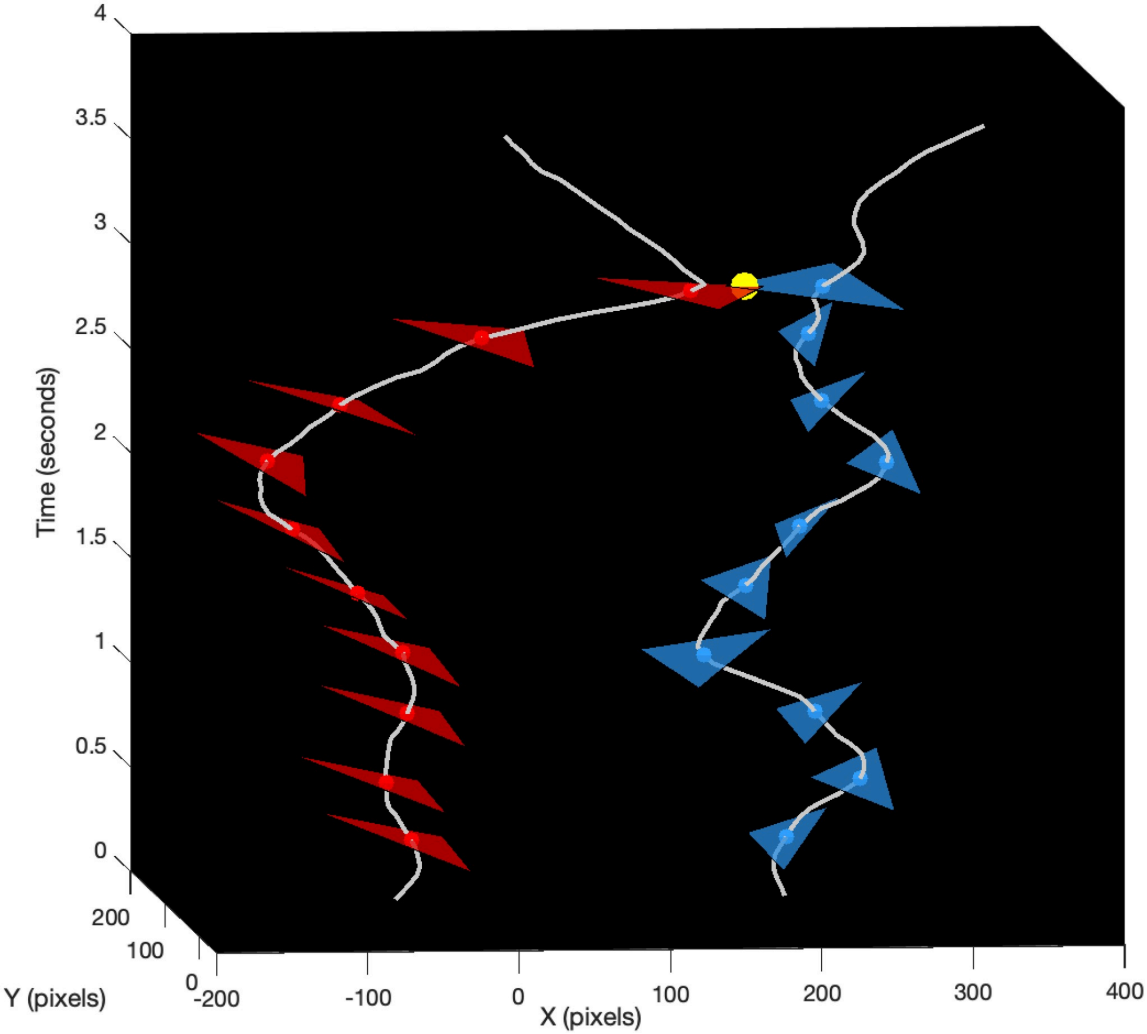
Illustrated motion scheme of frontal spaces. It describes movements of frontal spaces from the initial state to the hit state for Steffen (red) and Grumier (blue). Only a few triangles have been drawn for clarity. The worldlines (gray) represent the barycenters of the frontal spaces. The yellow ball represents the touch. It shows how Steffen won the touch with a direct attack.

The different phases of a duel can be determined from the analysis of the frontal spaces of the fencers. Fig 3 defines the frontal space as a triangular surface between the head, the sword hand and the top of the front foot. This strategic region in front of the fencer may be penetrated by the opponent to score the touch, while approaching it can be dangerous. It is closely related to the distance of danger discussed by Master Sicard [11], the former coach of the French Olympic team. Fig 4 graphically illustrates the motion scheme of frontal spaces as two transitions from the initial state to the hit state. It shows how Steffen won the touch with a direct attack.

**Fig 5:**
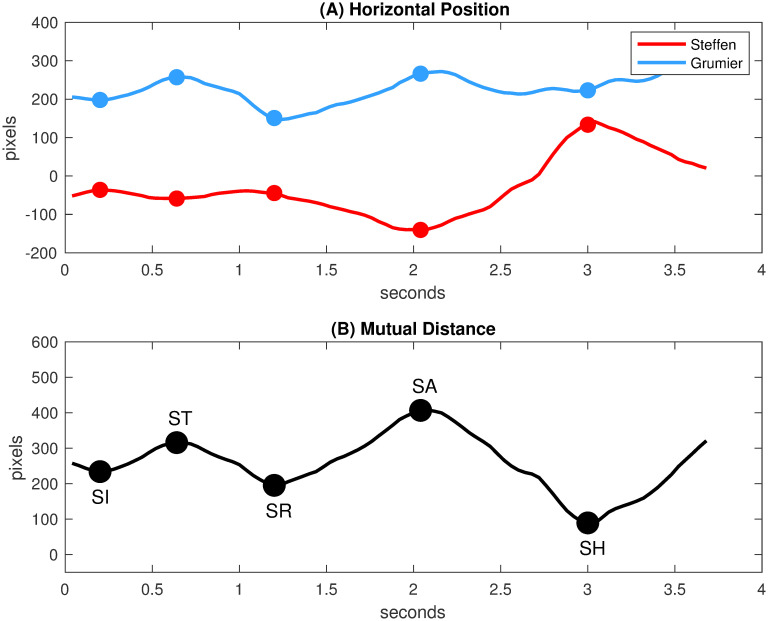
Analysis of the mutual distance. (A) Horizontal position of the barycenters of the frontal spaces, *X*_Steffen_(*t*) and *X*_Grumier_(*t*) respectively. (B) Mutual distance computed as |*X*_Steffen_(*t*) − *X*_Grumier_(*t*)|.

**Fig 6:**
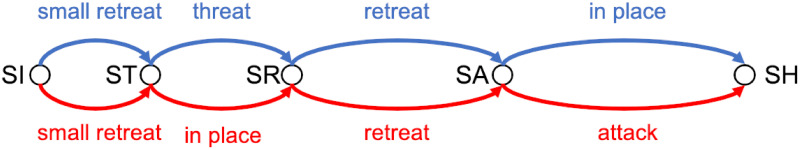
Motion scheme of the frontal spaces. The analysis of the mutual distance leads to a transition system for Steffen (red) and Grumier (blue). Each arrow represents a transition between two states realized by a frontal space.

As explained by Master Sicard [11], the changing distance between fencers is a crucial tactical element because its variations force participants to constantly adapt their position. The horizontal position in Fig 5A and the mutual distance in Fig 5B have been estimated from the barycenters of the frontal spaces of Steffen and Grumier. The critical points of the mutual distance in Fig 5B provide relevant information on the mutual actions of fencers. The events designated in the figure are the initial state [SI], a local maximum [ST], a local minimum [SR], a local maximum [SA] and the hit state [SH]. This alternation between minima and maxima reflects the variations in relative position between fencers as they approach or move away. Fig 6 shows the corresponding transition system that can be interpreted as follows:

[SI]→[ST] Fencers make a small retreat.

[ST]→[SR] Grumier performs a threatening action while Steffen stays in place.

[SR]→[SA] Fencers make a retreat.

[SA]→[SH] Steffen performs a direct attack while Grumier stays in place.

### Footwork

**Fig 7:**
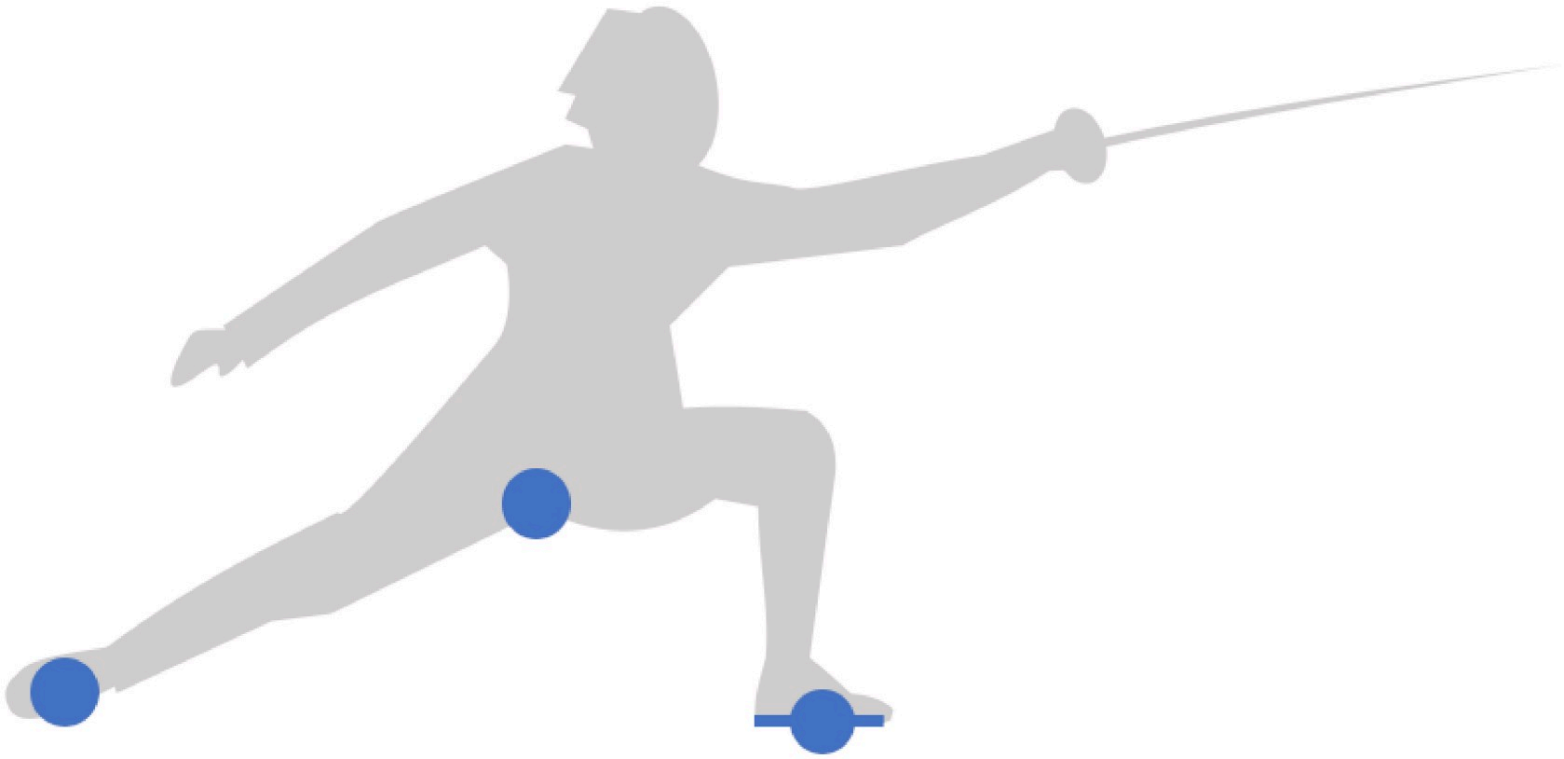
Footwork space. It is defined by the back foot, the crotch and the front foot. It captures important locomotion movements in the sagittal plane, such as step forward, step backward or lunge attack.

Footwork is produced by an atypical bipedal locomotion which is mainly influenced by the target at each moment of the duel. Fig 7 defines the footwork space by the back foot, the crotch and the front foot. The front foot inside the sagittal plane is represented by a line segment connecting the heel and the top, while the back foot across the sagittal plane is represented by a single point. The crotch is the pivot point between lower limbs, which provides an estimate of the fencer’s position relative to the ground. This footwork space captures important locomotion movements in the sagittal plane, such as step forward, step backward or lunge attack. The biomechanical properties of the feet for these types of movements are analyzed in an article by Trautmann et al. [12].

**Fig 8:**
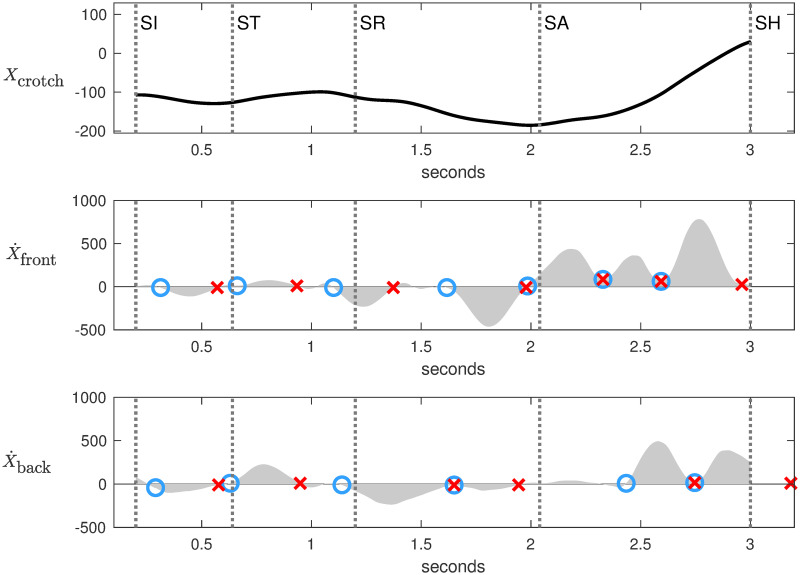
Footwork analysis of Steffen. *X*_crotch_ is horizontal position of the crotch. ${\dot{X}}_{\text{front}}$ is horizontal velocity of the front foot. ${\dot{X}}_{\text{back}}$ is horizontal velocity of the back foot. Each step corresponds to a velocity bump (gray area) delimited between a blue circle and a red cross.

**Fig 9:**
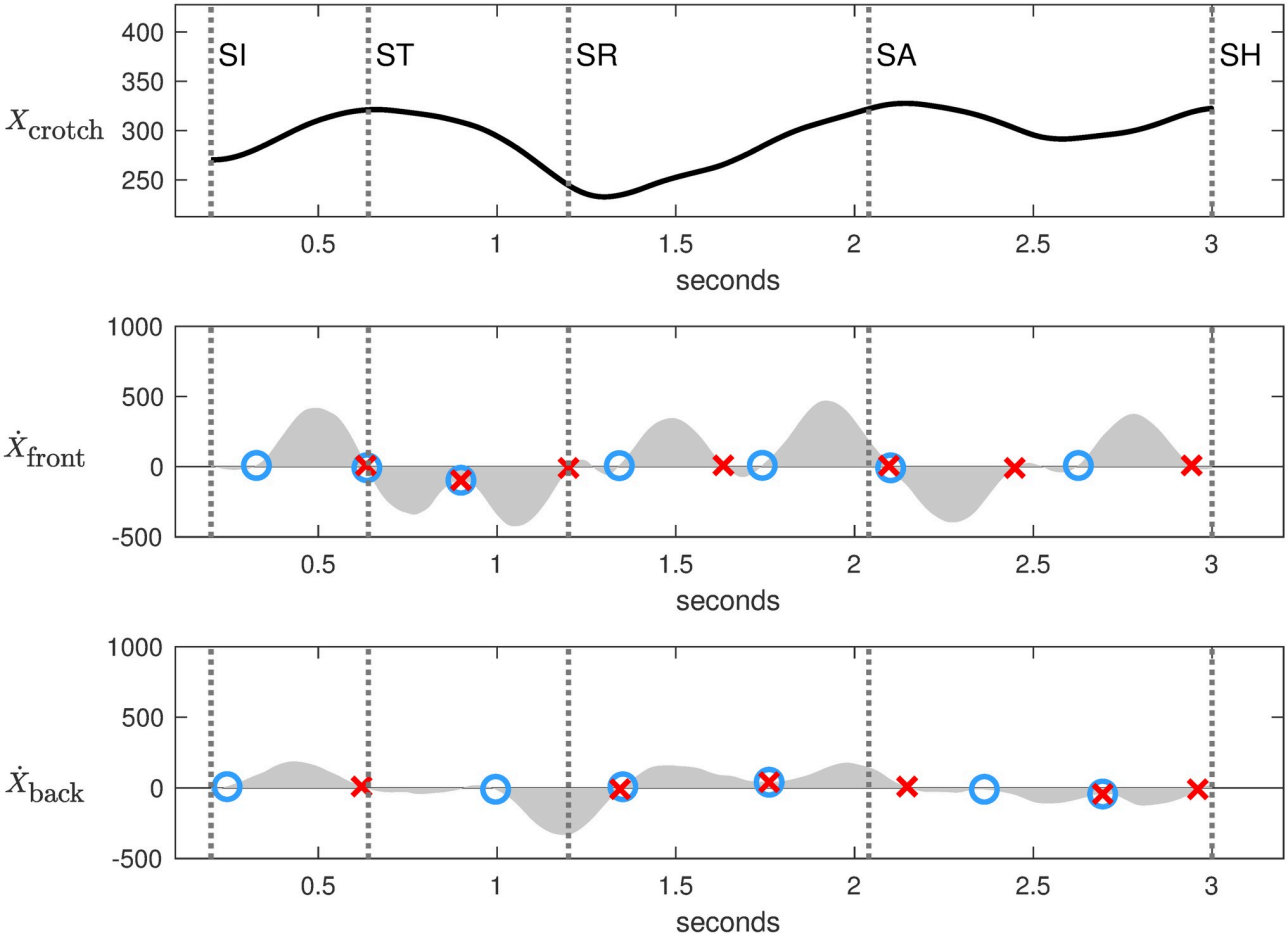
Footwork analysis of Grumier. *X*_crotch_ is horizontal position of the crotch. ${\dot{X}}_{\text{front}}$ is horizontal velocity of the front foot. ${\dot{X}}_{\text{back}}$ is horizontal velocity of the back foot. Each step corresponds to a velocity bump (gray area) delimited between a blue circle and a red cross.

Analysis of horizontal components in the footwork space reveals how fencers move along the piste to control the mutual distance. Three observables were measured: horizontal position of the crotch *X*_crotch_, horizontal velocity of the front foot ${\dot{X}}_{\text{front}}$ and horizontal velocity of the back foot ${\dot{X}}_{\text{back}}$. The velocity of the front foot was estimated as the average velocity between the heel and the top of the foot. The time derivatives were computed with a Savitzky-Golay filter to smooth undesirable noise. Figs 8 and 9 represent the three observables for Steffen and Grumier respectively.

A custom algorithm was developed in Matlab to identify the horizontal movements of the feet from the ${\dot{X}}_{\text{front}}$ and ${\dot{X}}_{\text{back}}$ velocities curves of Figs 8 and 9. Each step corresponds to a velocity bump (gray area) delimited by an initial zero velocity (blue circle) and a final zero velocity (red cross). A positive bump corresponds to a step forward for Steffen and a step backward for Grumier, and vice versa for a negative bump. A manually adjustable threshold was used to ignore bumps of small amplitude. A red cross inside a blue circle characterizes two consecutive synchronized steps. This method resulted in the following analysis of footwork:

[SI]→[ST] Fencers make a small retreat. ${\dot{X}}_{\text{front}}$ and ${\dot{X}}_{\text{back}}$ show that they take a step backward for each foot. Grumier’s movements are more pronounced than those of Steffen.

[ST]→[SR] Grumier performs a threatening action while Steffen stays in place. ${\dot{X}}_{\text{front}}$ shows that Grumier executes two consecutive synchronized steps forward initiated at the end of the small retreat. ${\dot{X}}_{\text{back}}$ shows that Grumier first advances the front foot but not the back foot, then he advances both feet to push forward. Meanwhile, Steffen stays in place with weaker foot movements.

[SR]→[SA] Fencers make a retreat. ${\dot{X}}_{\text{front}}$ and ${\dot{X}}_{\text{back}}$ show that they use the same technique of taking two steps backward for each foot. More precisely, ${\dot{X}}_{\text{front}}$ have two spaced bumps of high amplitude while ${\dot{X}}_{\text{back}}$ have two consecutive synchronized bumps of lower amplitude. This indicates that the front feet make impulses backward while the back feet are coordinated to follow.

[SA]→[SH] Steffen performs a direct attack while Grumier stays in place. ${\dot{X}}_{\text{front}}$ shows that Steffen executes three consecutive synchronized steps forward initiated at the end of the retreat. ${\dot{X}}_{\text{back}}$ shows that Steffen first advances the front foot but not the back foot, then he advances both feet to push forward to the hit. In contrast, Grumier is hesitant, ${\dot{X}}_{\text{front}}$ shows that the front foot takes a step forward followed by a step backward while ${\dot{X}}_{\text{back}}$ shows a back foot with a lower amplitude.

**Fig 10:**
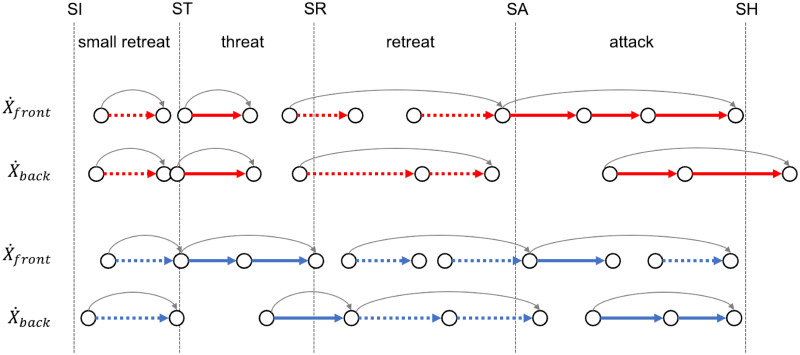
Motion scheme of footwork. The velocity bumps of Figs 8 and 9 determine a transition system. Each thick arrow represents a foot step forward (solid arrow) or backward (dotted arrow) for Steffen (red) and Grumier (blue). Each thin arrow (gray) represents a fencing action (small retreat, threat, retreat, attack).

This scenario based on velocity bumps determines the motion scheme in Fig 10. It is interesting to note that Steffen’s attack and Grumier’s threat have similar constructions. In each case, the fencer leaves after a retreat, first advancing the front foot but not the back foot, then advancing both feet to push forward. In addition, the front foot moves with consecutive synchronized steps. Such an organized chain of actions is probably the result of an assortment of automatisms learned during the training. Master Sicard [11] explains that automatisms aim to reduce the time cost of the action. Fencers have so little time to make decisions that action is difficult to distinguish from intention. This supports the fact that consecutive synchronized steps minimize the time between steps of the front foot during offensive actions. This suggests a strategic anticipation of a sequence of actions rather than of a single action. In this way, fencing is comparable to a game of chess.

### Kinematic model

**Fig 11:**
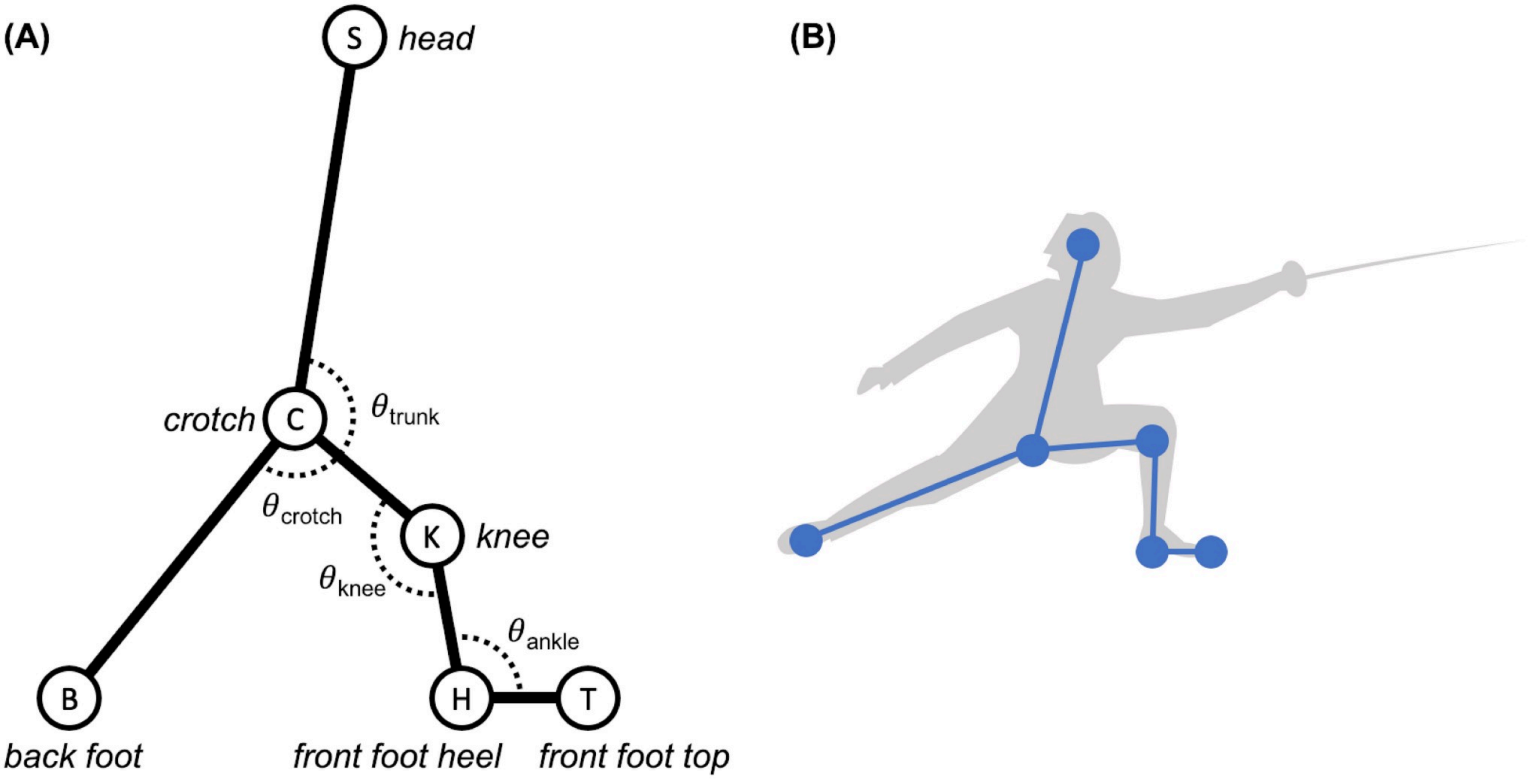
Kinematic model. **(A)** It is composed of segments and joints. CS = trunk, CB = rear lower limb, CK = front thigh, KH = front leg, HT = front foot, $\theta_{\text{trunk}}=\hat{SCK}$, $\theta_{\text{crotch}}=\hat{KCB}$, $\theta_{\text{knee}}=\hat{HKC}$, $\theta_{\text{ankle}}=\hat{KHT}$. **(B)** Implementation of this model based on video tracking.

Physiologically, human motion is the result of complex movement patterns induced by muscle forces and joint moments. Rigid body models based on limb segments and their joints reduce the complexity of human motion to a small set of observables. Fig 11 illustrates a kinematic model for Steffen and Grumier composed of segments and joints. The segments considered are the trunk, the rear lower limb, the front thigh, the front leg, the front foot. The joints considered are the trunk angle *θ*_trunk_, the crotch angle *θ*_crotch_, the knee angle *θ*_knee_ in flexion and extension, the ankle angle *θ*_ankle_ in dorsiflexion and plantarflexion. The time derivatives were computed with a Savitzky-Golay filter to smooth undesirable noise. This model captures relevant information although the trunk and rear lower limb are not strictly limb segments. Accurate measurements on rigid body models are discussed by Andriacchi et al. [13]. The kinematic models of the two fencers are comparable because Steffen, 189cm, 84kg, and Grumier, 188cm, 82kg have similar corpulence (source of the metrics: Wikipedia).

#### Rigidity

**Fig 12:**
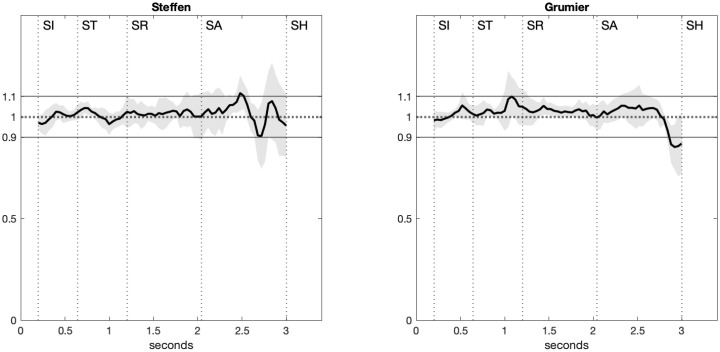
Average relative length. The average relative length of the five segments of the kinematic model is in the range 1 ± 0.1 except for a few peaks of standard deviation during offensive actions.

In order to check that segments of the kinematic model are approximately rigid, the relative length of each segment was measured as

$$\tilde{L_{k}}(t)=\frac{L_{k}(t)}{\left\langle L_{k}\right\rangle }$$

where *L*_*k*_(*t*) is the length of segment *k* at time *t* and 〈*L*_*k*_〉 is its length averaged over time. By definition, $\tilde{L_{k}}=1$ for a perfectly rigid segment. The relative lengths of the five segments of the kinematic model were averaged and plotted in Fig 12 as a solid curve surrounded by standard deviation area. The average relative length is in the range 1 ± 0.1 except for a few peaks of standard deviation during offensive actions, namely the attack ([SA]→[SH] for Steffen and Grumier) and the threat ([ST]→[SR] for Grumier).

#### Trunk and crotch angles

**Fig 13:**
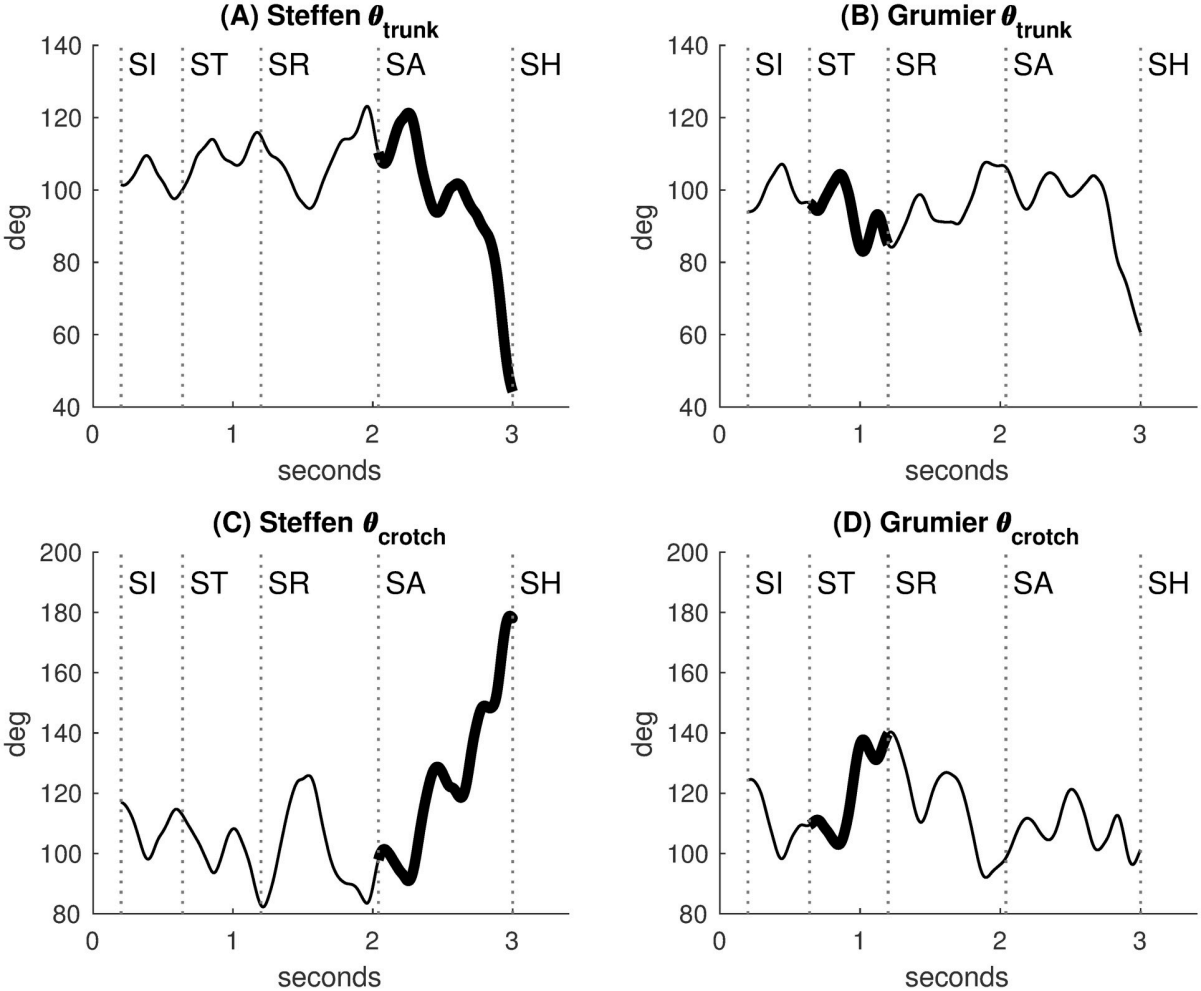
Trunk and crotch angles. **(A)** The trunk angle dramatically decreases during the Steffen’s attack. **(B)** The trunk angle decreases during the Grumier’s threat. **(C)** The crotch angle dramatically increases during the Steffen’s attack. **(D)** The crotch angle increases during the Grumier’s threat.

The angles *θ*_trunk_ and *θ*_crotch_ measure trunk and crotch movements respectively. When the fencer is upright, *θ*_trunk_ is open and *θ*_crotch_ is closed. When the fencer lies down, *θ*_trunk_ tends to decrease and *θ*_crotch_ tends to increase. Indeed, during the Steffen’s attack [SA]→[SH], *θ*_trunk_ dramatically decreases to a minimum (Fig 13A) and *θ*_crotch_ dramatically increases to a maximum (Fig 13C). To a lesser extent, during the Grumier’s threat [ST]→[SR], *θ*_trunk_ decreases (Fig 13B) and *θ*_crotch_ increases (Fig 13D).

#### Trunk and crotch angular velocities

**Fig 14:**
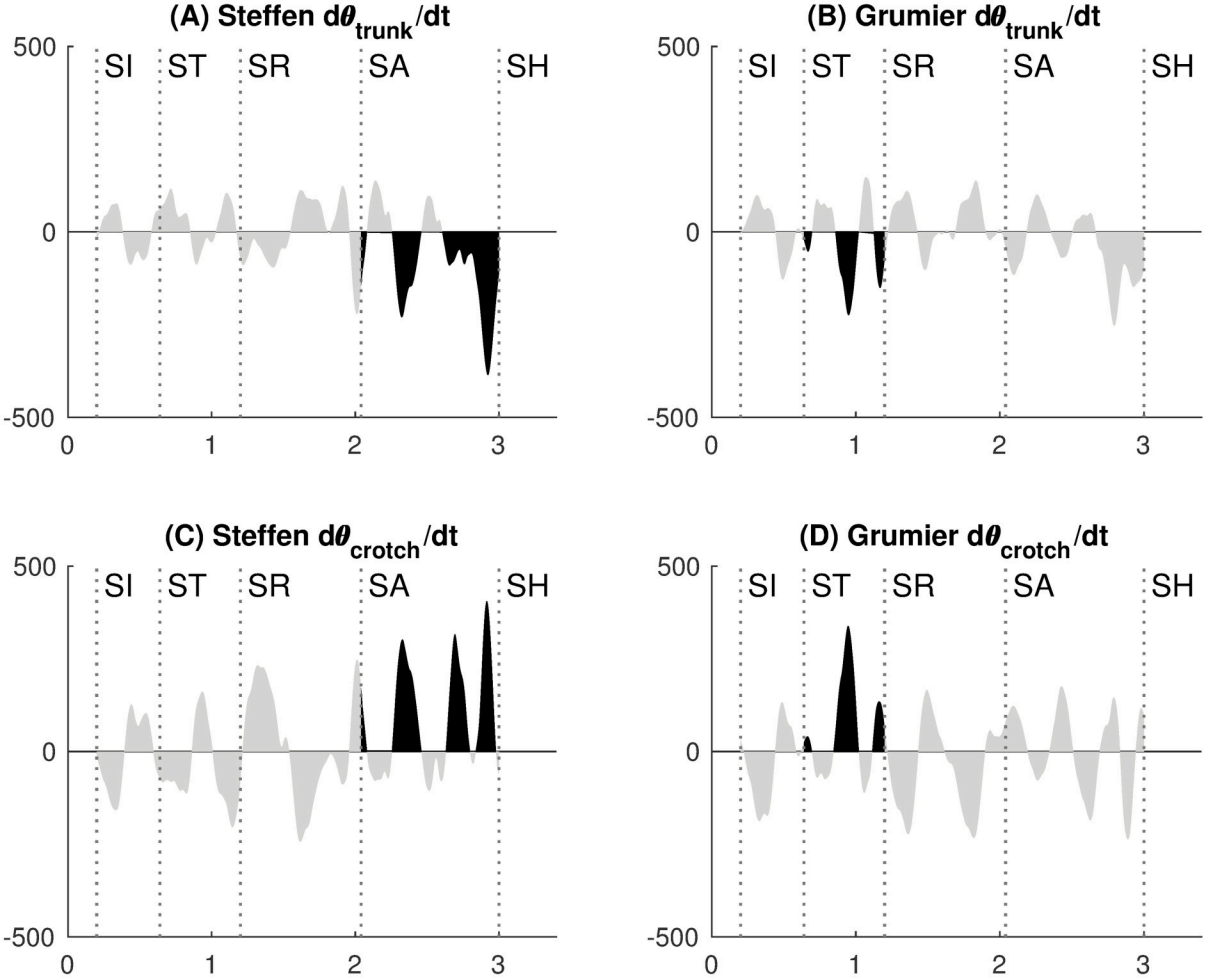
Trunk and crotch angular velocities. **(A)** Negative velocity peaks when closing the trunk during the Steffen’s attack. **(B)** Negative velocity peaks when closing the trunk during the Grumier’s threat. **(C)** Positive velocity peaks when opening the crotch during the Steffen’s attack. **(D)** Positive velocity peaks when opening the crotch during the Grumier’s threat.

The angular velocities ${\dot{\theta }}_{\text{trunk}}$ and ${\dot{\theta }}_{\text{crotch}}$ are represented in Fig 14. Each bump in angular velocity characterizes a monotonic movement. Positive bumps correspond to the opening of trunk and crotch angles. Negative bumps correspond to the closing of trunk and crotch angles.

For Steffen, dominant velocity peaks occur during the attack [SA]→[SH] when closing the trunk (Fig 14A) and opening the crotch (Fig 14C). Similarly for Grumier, dominant velocity peaks occur during the threat [ST]→[SR] when closing the trunk (Fig 14B) and opening the crotch (Fig 14D).

#### Knee and ankle angles

**Fig 15:**
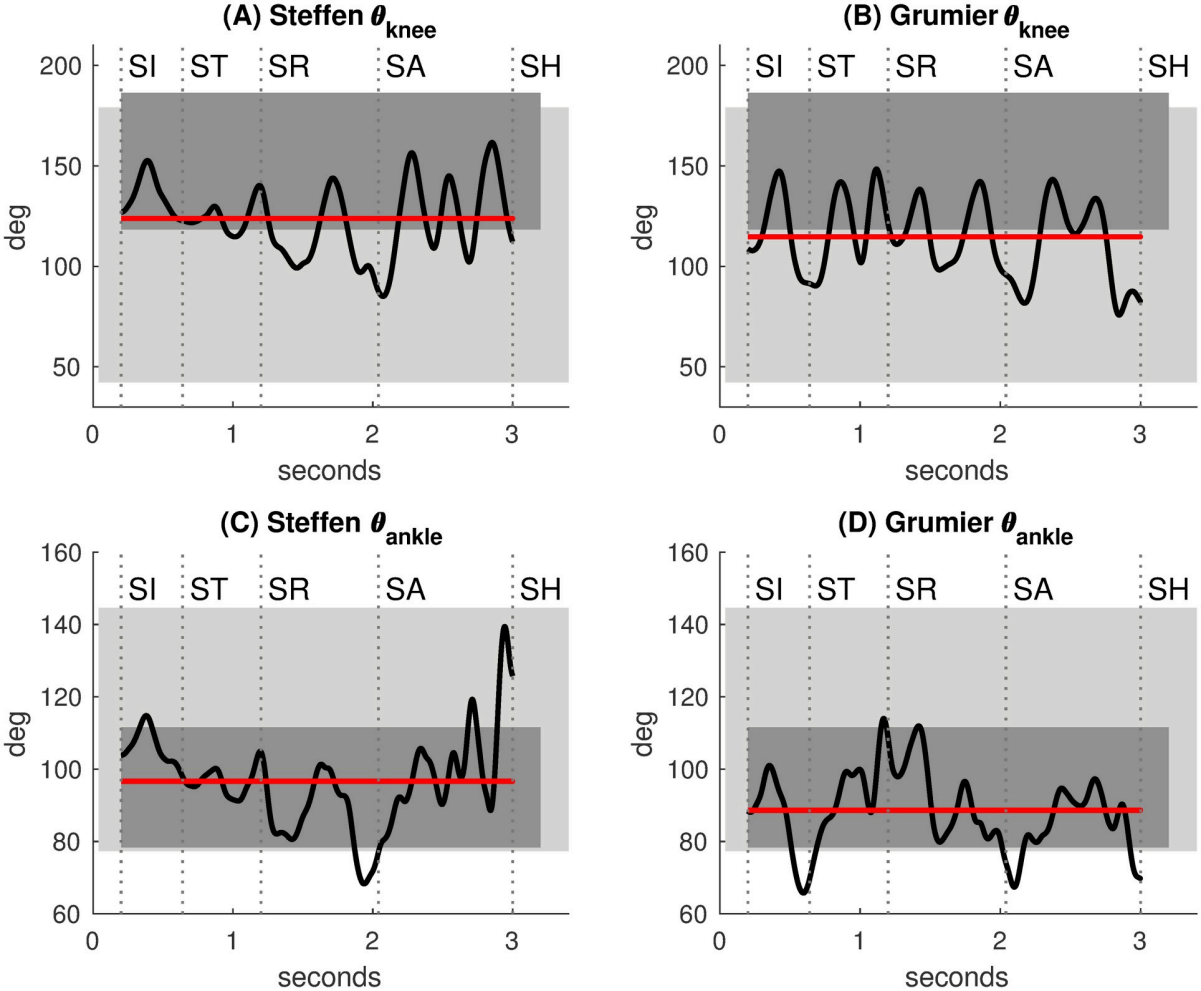
Knee and ankle angles. Light rectangles: ActiveROM, active range of motion from Faisal et al. [14]. Dark rectangles: WalkingROM, walking range of motion from Mentiplay et al. [15]. Red lines: average values of the angles. **(A,B)** The knee angles of Steffen and Grumier vary around the lower limit of WalkingROM, fencers bend their knees. **(C,D)** The ankle angles of Steffen and Grumier are mainly within WalkingROM.

The angles *θ*_knee_ and *θ*_ankle_ measure knee and ankle movements respectively. These are represented in Fig 15. The light rectangles represent ActiveROM, the normative values of active range of motion for human joints published by Faisal et al. [14] (males 20-44 years). The dark rectangles represent WalkingROM, the normative values of walking range of motion for human joints published by Mentiplay et al. [15] (maximum range of motion for speeds 1.40–1.60 m/s in normal and dynamic walking). Standard deviation fluctuations were ignored for the sake of simplification.

For Steffen and Grumier (Fig 15A and 15B), *θ*_knee_ is within ActiveROM and below the upper limit of WalkingROM. However, *θ*_knee_ significantly exceeds the lower limit of WalkingROM for pronounced knee flexion. The average value of *θ*_knee_ (red line) is close to the lower limit of WalkingROM. This indicates that fencers bend their knees during the duel. This practice is well known in fencing to better distribute the body weight on the feet.

For Steffen and Grumier (Fig 15C and 15D), the average value of *θ*_ankle_ is within WalkingROM. It significantly increases for Steffen during the touch.

#### Knee and ankle angular velocities

**Fig 16:**
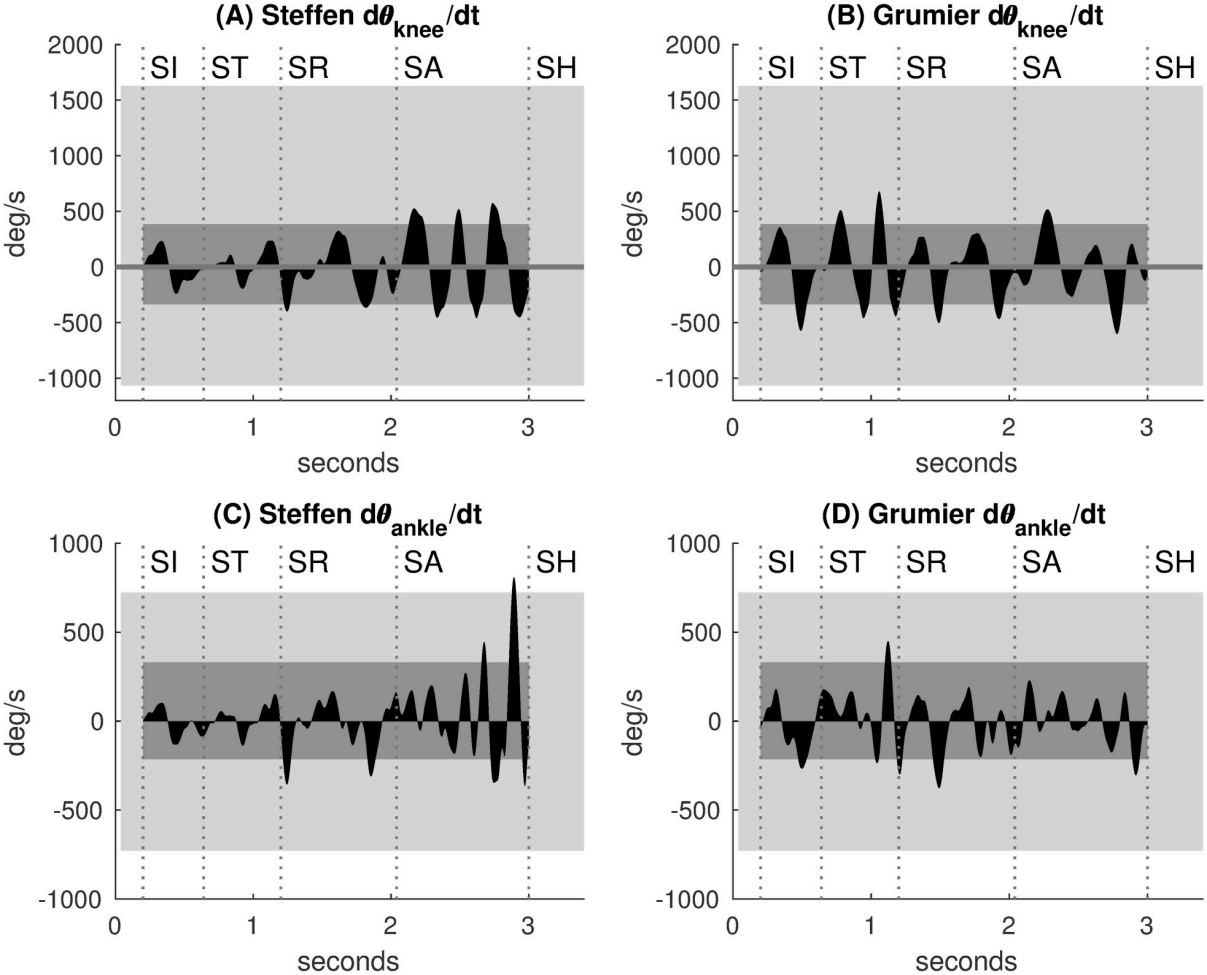
Knee and ankle angular velocities. Light rectangles: MaxAV, maximum angular velocities for sport from Jessop et al. [16]. Dark rectangles: WalkingAV, angular velocities during walking from Mentiplay et al. [15]. **(A,B,C,D)** The knee and ankle angular velocities of Steffen and Grumier are mainly within WalkingAV and enhanced during their offensive actions.

The angular velocities ${\dot{\theta }}_{\text{knee}}$ and ${\dot{\theta }}_{\text{ankle}}$ are represented in Fig 16. Each bump in angular velocity characterizes a monotonic movement. Positive bumps correspond to knee extension and ankle plantarflexion. Negative bumps correspond to knee flexion and ankle dorsiflexion. The light rectangles represent MaxAV, the normative values of maximum angular velocities in flexion and extension actions for sport published by Jessop et al. [16] (unrestricted condition). The dark rectangles represent WalkingAV, the normative values of angular velocities during walking published by Mentiplay et al. [15] (speeds 1.40–1.60 m/s in normal and dynamic walking). Standard deviation fluctuations were ignored for the sake of simplification.

For Steffen and Grumier (Fig 16), ${\dot{\theta }}_{\text{knee}}$ and ${\dot{\theta }}_{\text{ankle}}$ are mainly within WalkingAV, and thus also within MaxAV. Velocity peaks of knee and ankle are enhanced when fencers propel themselves forward during Steffen’s attack and Grumier’s threat.

#### Motion scheme of the kinematic model

**Fig 17:**
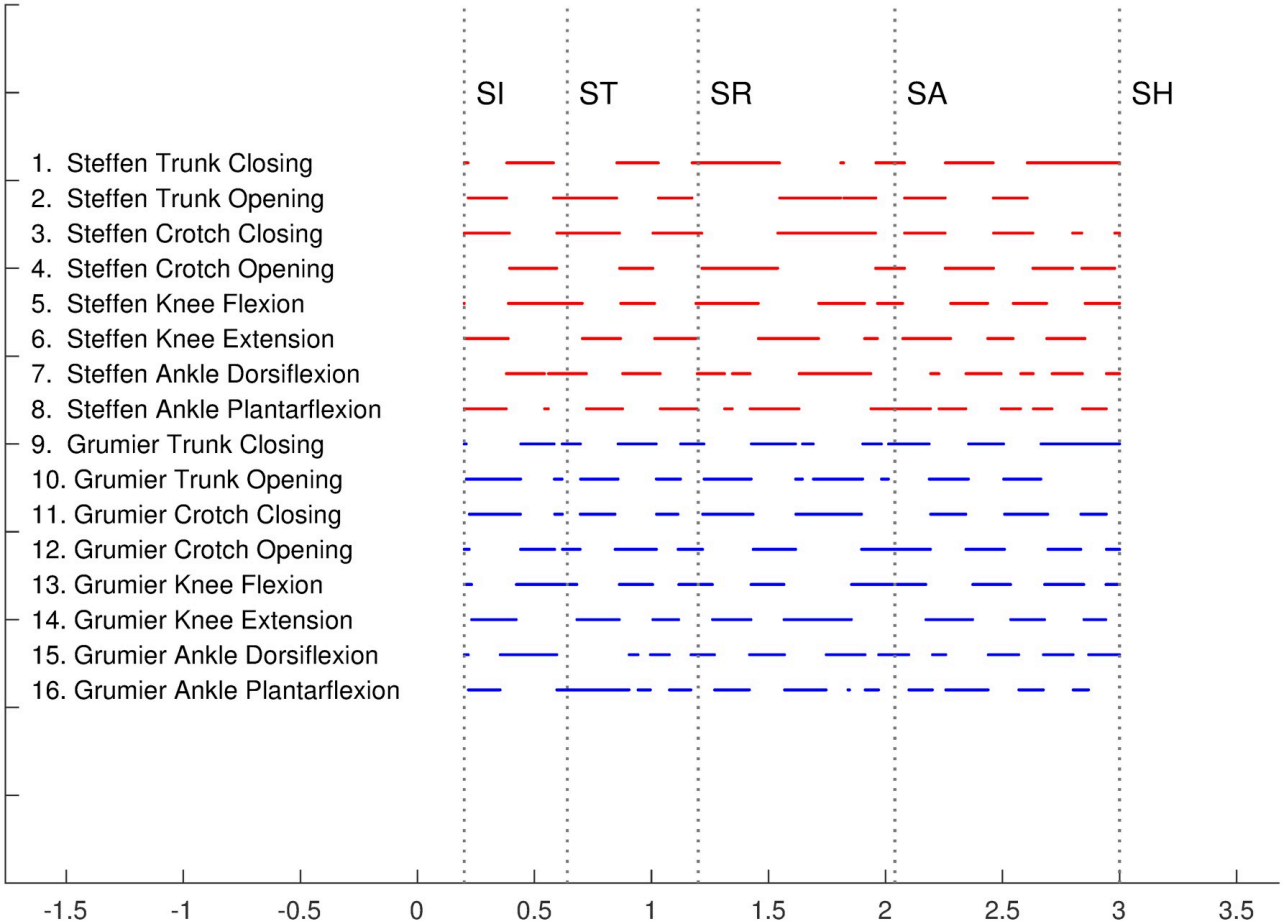
Motion scheme of the kinematic model. Each segment represents a monotonic movement determined by an angular velocity bump in Figs 14 and 16.

The monotonic movements of trunk closing/opening, crotch closing/opening, knee flexion/extension, ankle dorsiflexion/plantarflexion are summarized by the motion scheme in Fig 17. Each segment on the diagram corresponds to an angular velocity bump, which represents a monotonic joint movement in the kinematic model. Each *k*-th row in the diagram is encoded as a characteristic function *v*_*k*_(*t*) such that *v*_*k*_(*t*) = 1 when a segment of the row is defined at *t* and *v*_*k*_(*t*) = 0 otherwise.

**Fig 18:**
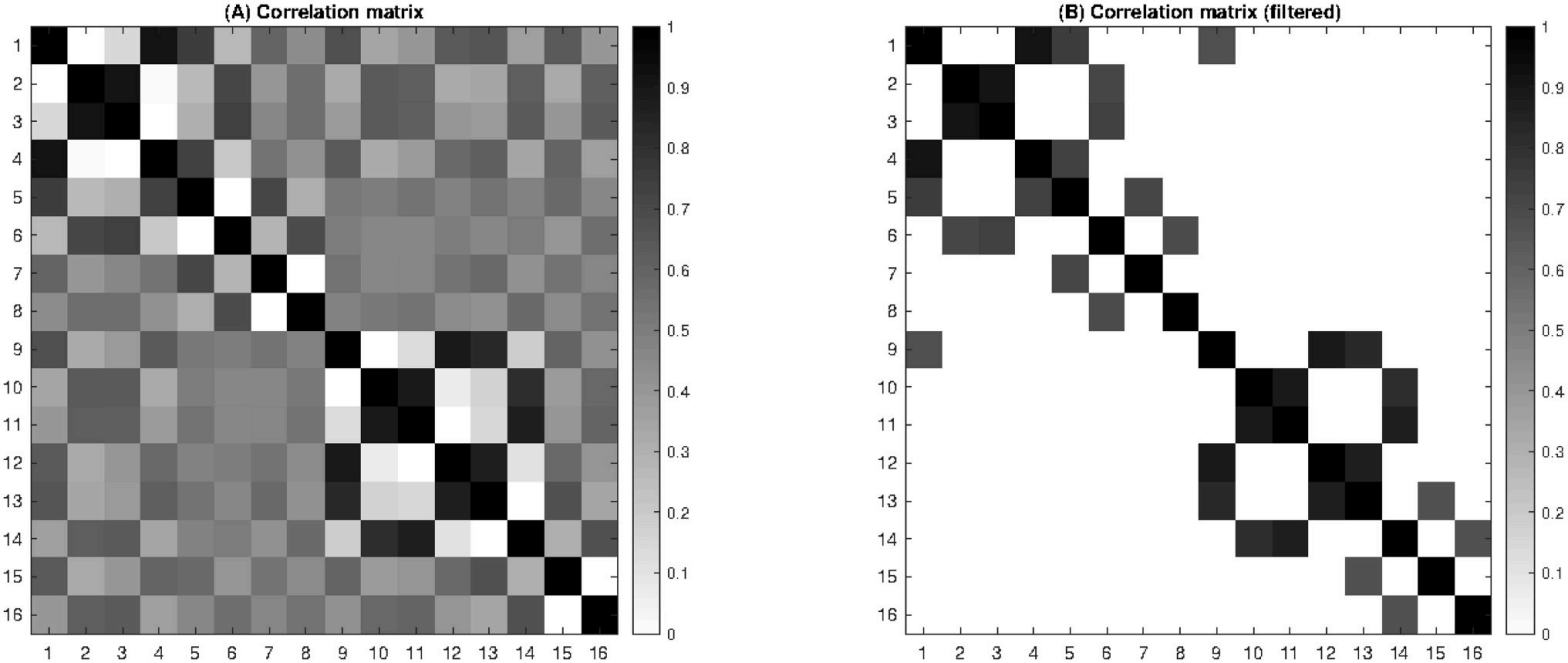
Correlation matrix of the motion scheme of the kinematic model. **(A)** Each coefficient *c*_*ij*_ measures the correlation between rows *i* and *j* in the motion scheme of Fig 17. **(B)** The same matrix filtered by a threshold at 0.66 (∼ 2/3) reveals non-trivial coordination patterns common to Steffen and Grumier.

Fig 18A shows the correlation matrix of the motion scheme of the kinematic model. The coefficients of the correlation matrix are given by

$$c_{ij}=\frac{v_{i}\cdot v_{j}}{\left\|v_{i}\|\|v_{j}\right\|}$$

where the dot product and the norm are canonical. The correlation coefficients *c*_*ij*_ are normalized between 0 and 1. By construction, the matrix is symmetric and its diagonal coefficients are equal to 1. The matrix has trivial zeros for opposite movements, such as knee flexion and extension.

In order to simplify the correlation matrix, Fig 18B shows the coefficients filtered by a threshold at 0.66 (∼ 2/3). This reveals non-trivial coordination patterns common to Steffen and Grumier:

- *c*_3,2_ and *c*_11,10_: crotch closing & trunk opening
- *c*_4,1_ and *c*_9,12_: crotch opening & trunk closing
- *c*_5,1_ and *c*_9,13_: knee flexion & trunk closing
- *c*_5,4_ and *c*_13,12_: knee flexion & crotch opening
- *c*_6,2_ and *c*_14,10_: knee extension & trunk opening
- *c*_6,3_ and *c*_14,11_: knee extension & crotch closing
- *c*_7,5_ and *c*_15,13_: ankle dorsiflexion & knee flexion
- *c*_8,6_ and *c*_16,14_: ankle plantarflexion & knee extension
- *c*_9,1_: Steffen and Grumier simultaneously closing the trunk

Such patterns are related to motor control, neuromuscular control, mechanisms of stability, proprioception, that could be investigated in future work. It is interesting to note that the last pattern is interactive, which suggests that the two fencers can synchronize their dynamic posture in action.

### Lunge

The lunge is a crucial attack technique in which the fencer quickly propels himself forward and pushes the sword towards the target. The article on biomechanics of fencing sport by Chen et al. [17] reports normative values from existing studies for the peak velocity at mass center (1.72 m/s), weapon (2.49 m/s) and front foot (4.10 m/s) during the execution of a lunge attack with épée. More specific measurements are given by Gutierrez-Davila et al. [18] using force platforms and infrared video cameras for the maximum velocity at mass center (1.93 m/s), weapon (2.55 m/s) and front foot (4.56 m/s). In this section, Steffen’s lunge attack is compared to the values given by Chen et al. [17] and Gutierrez-Davila et al. [18].

**Fig 19:**
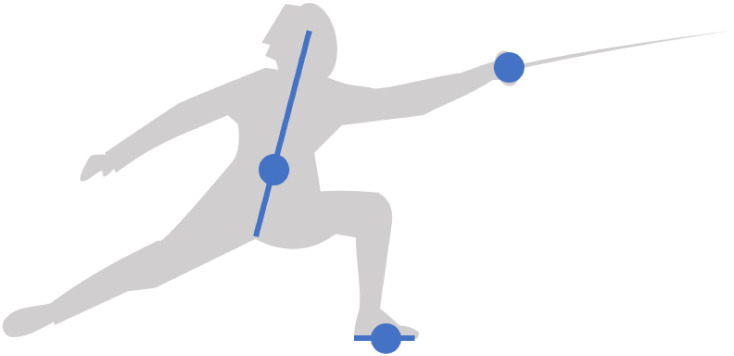
Lunge space. It is defined by the body center (at $\frac{1}{3}$ of the trunk segment), the sword hand and the front foot (in the middle of the foot segment). These three points of interest are comparable to the mass center, the weapon and the front foot studied by Chen et al. [17] and Gutierrez-Davila et al. [18].

Fig 19 defines the lunge space by the body center, the sword hand and the front foot. These three points of interest are comparable to those of the two articles cited above. The body center is defined at $\frac{1}{3}$ of the trunk segment from the crotch to the head, at the height of the umbilicus in reference to Plate 1 of Netter’s Atlas of Human Anatomy [19]. The body center plays a similar role to the mass center in that it gives an estimate of the fencer’s velocity in the sagittal plane. The sword hand coincides with the épée handle, which gives an approximation of the weapon velocity. The front foot velocity is evaluated in the middle between the heel and the top of the foot.

#### Characterization

**Fig 20:**
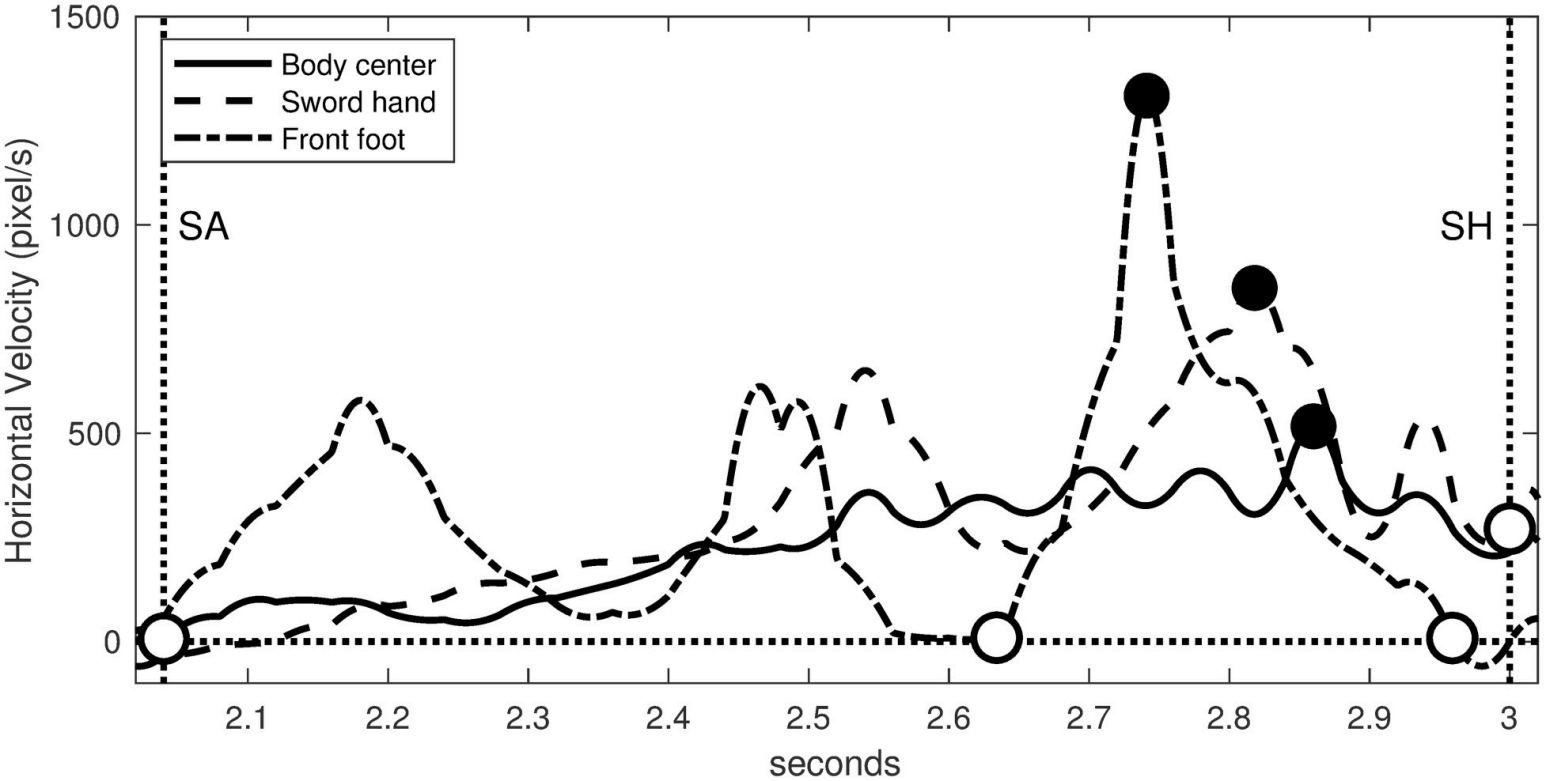
Characterization of Steffen’s lunge attack. Horizontal velocities are measured at the body center (solid line), sword hand (dashed line) and front foot (dash-dot line). Decisive movements are delimited by black dots (maximum velocity peaks) and white dots.

Fig 20 represents the horizontal velocities of Steffen’s lunge attack, which satisfy the following scenario:

- **Body center**: acceleration phase from zero to maximum velocity peak.
- **Sword hand**: thrust of sword from maximum velocity peak to touché.
- **Front foot**: step forward through a velocity bump.

**Fig 21:**
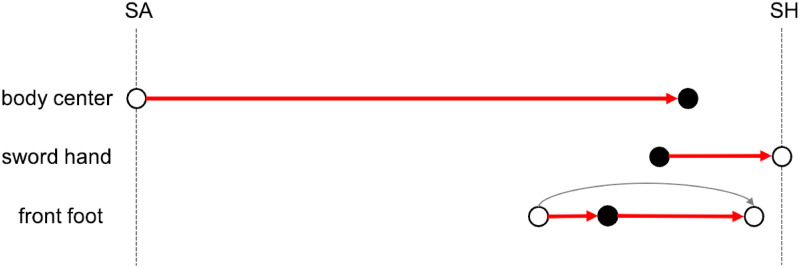
Motion scheme of Steffen’s lunge attack. The body center arrow describes the acceleration phase. The sword hand arrow describes the thrust to the touché. The front foot arrow describes the propulsion of one step forward. Maximum velocity peaks are indicated by black dots.

This scenario is consistent with the three velocity curves given by Gutierrez-Davila et al. [18]. It determines the motion scheme given in Fig 21. Each arrow corresponds to a decisive movement of the body center, the sword hand and the front foot respectively.

#### Comparison

**Fig 22:**
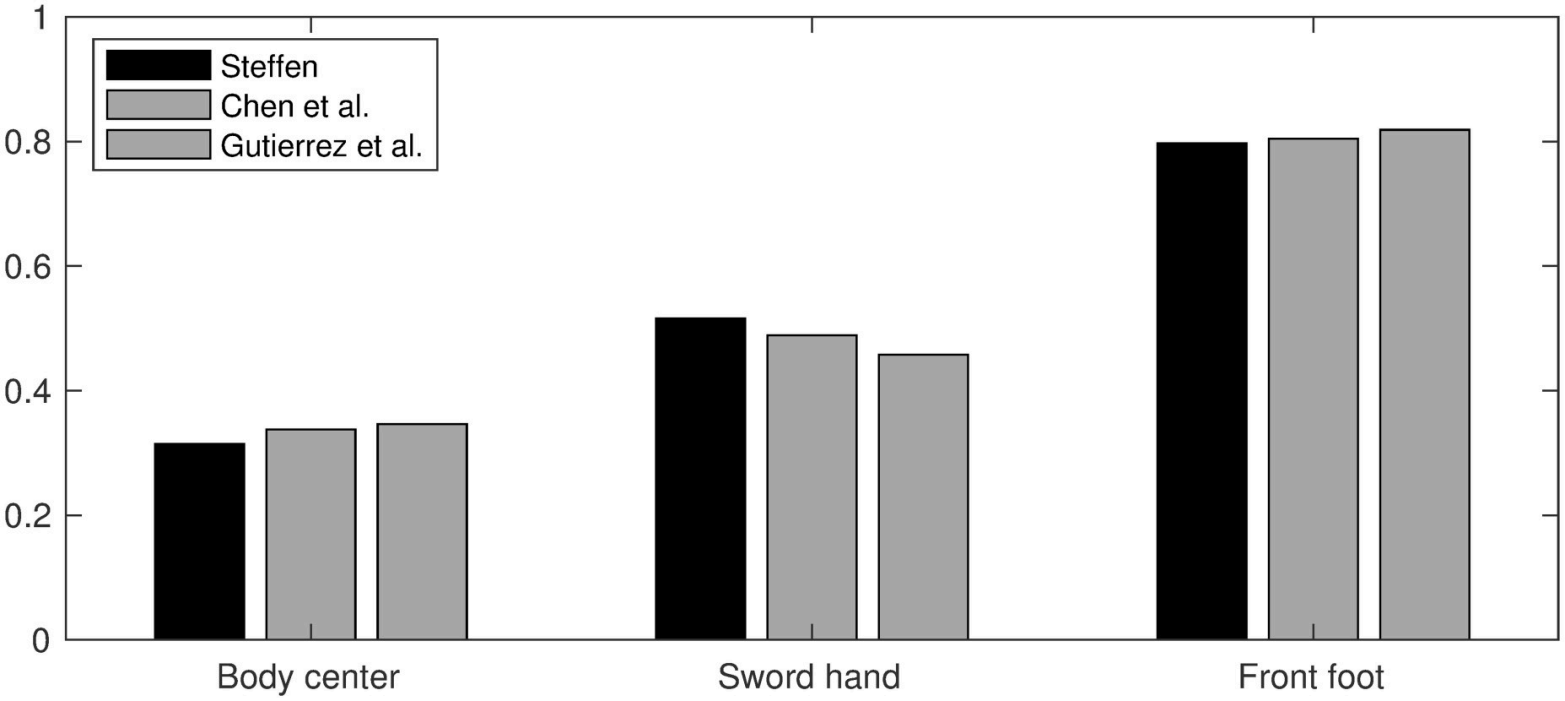
Normalized velocities. Steffen’s normalized velocities match those of Chen et al. [17] at 97% and Gutierrez-Davila et al. [18] at 94%, which is good accuracy.

The Steffen velocity peaks in Fig 20 (pixel/s) are reported in Table 1 along with the normative values (m/s) indicated by Chen et al. [17] and Gutierrez-Davila et al. [18]. Each triplet of velocities is encoded by a vector **v** = (*v*_center_, *v*_hand_, *v*_foot_) and then normalized as **v**/‖**v**‖. Fig 22 compares the normalized vector components by juxtaposing bar charts. The Steffen’s normalized vector components match those of Chen et al. [17] at 97% and Gutierrez-Davila et al. [18] at 94%, which is good accuracy.

**Table 1:** Peak velocities.

	Steffen (pixels/s)	Chen (m/s)	Gutierrez-Davila (m/s)
body center	517	1.72	1.93
sword hand	849	2.49	2.55
front foot	1310	4.10	4.56

### Free hand

The role of the free hand is rarely mentioned in the scientific literature. In 1836, Chatauvillard was the first to codify the art of the duel in his *Essai sur le Duel* [20]. He banned the use of the free hand which, in previous centuries, could grasp the opponent’s sword hand or hold a second weapon or shield. In modern fencing, the free hand is ungloved and held aside most of the time. Although the free hand is not used against the opponent, it plays a crucial role in controlling the body at all times. It must be protected against dangerous actions of the opponent in order to avoid injuries. It is also involved in the balance of the body during the lunge.

**Fig 23:**
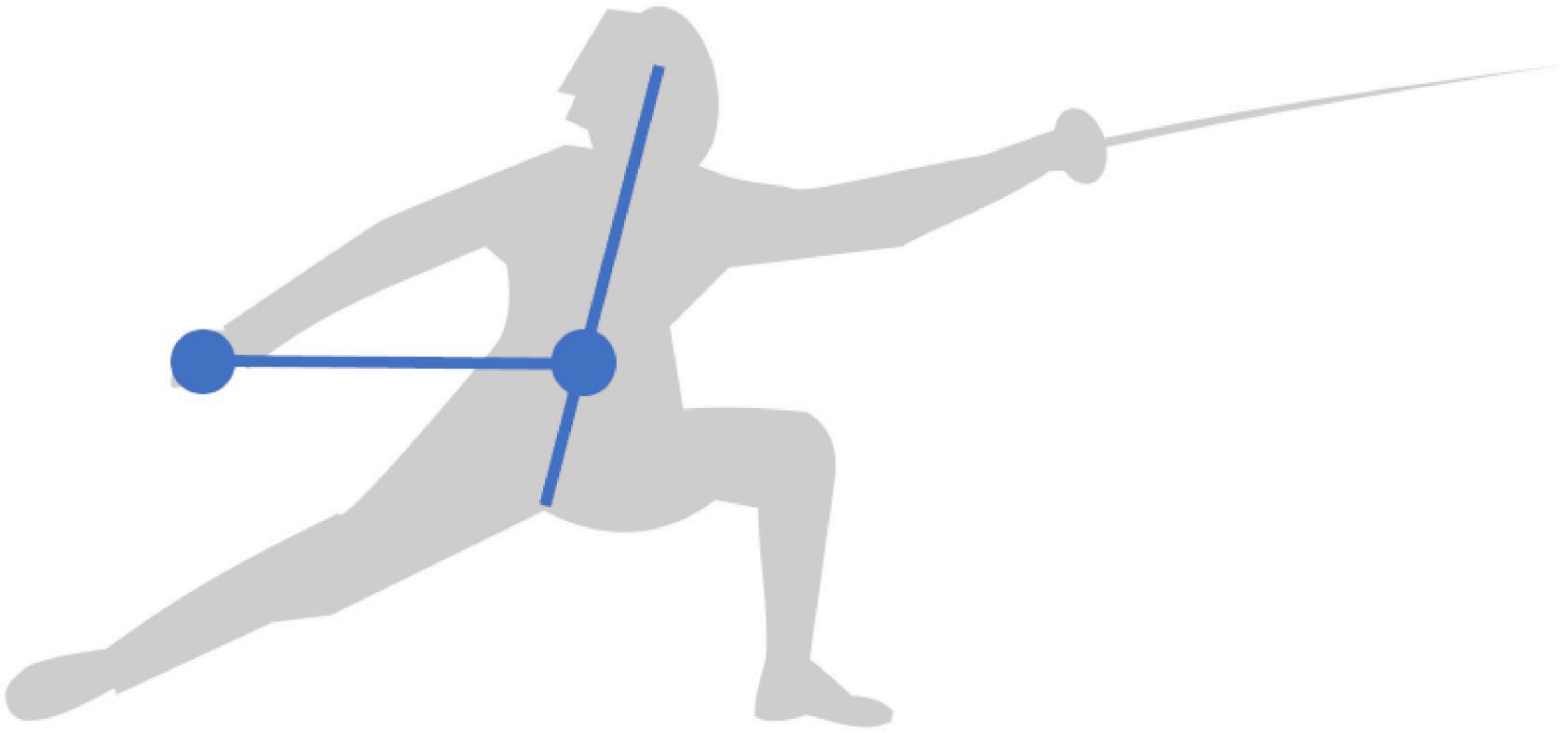
Free hand space. It is defined by a line segment between the free hand and the body center. The free hand movement is evaluated relative to the body center.

**Fig 24:**
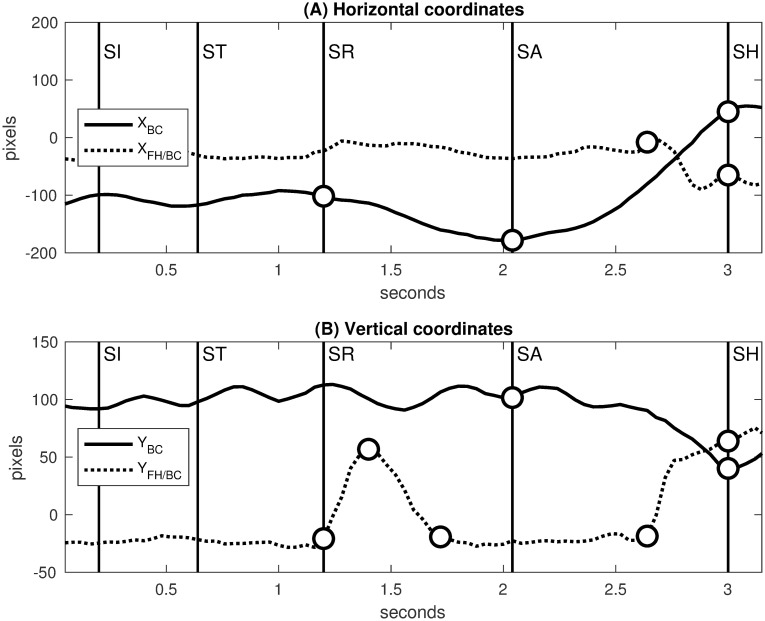
Trajectories of Steffen’s body center and free hand. Decisive movements are delimited by white dots (variation changes). **(A)** Horizontal coordinates of body center (*X*_BC_, solid line) and free hand relative to body center (*X*_FH/BC_, dotted line). **(B)** Vertical coordinates of body center (*Y*_BC_, solid line) and free hand relative to body center (*Y*_FH/BC_, dotted line).

Fig 23 defines the free hand space by a line segment between the free hand and the body center estimated as previously in Fig 19. In this way, the free hand movement is evaluated relative to the body center. Fig 24 shows the trajectories of Steffen’s body center and free hand decomposed along the horizontal and vertical axes.

During the retreat [SR]→[SA], the body center moves horizontally backward (Fig 24A) while the free hand moves vertically up and down (Fig 24B). This protective gesture occurs at the end of Grumier’s threat when the mutual distance reaches a local minimum at [SR] (Fig 5).

At the end of the lunge attack [SA]→[SH], Steffen moves the body center forward and downward (Fig 24A and 24B) while the free hand is thrown backward and upward (Fig 24A and 24B). This balancing gesture occurs at the end of Steffen’s attack using the free hand as a counterweight to the propelling movement towards the target.

**Fig 25:**
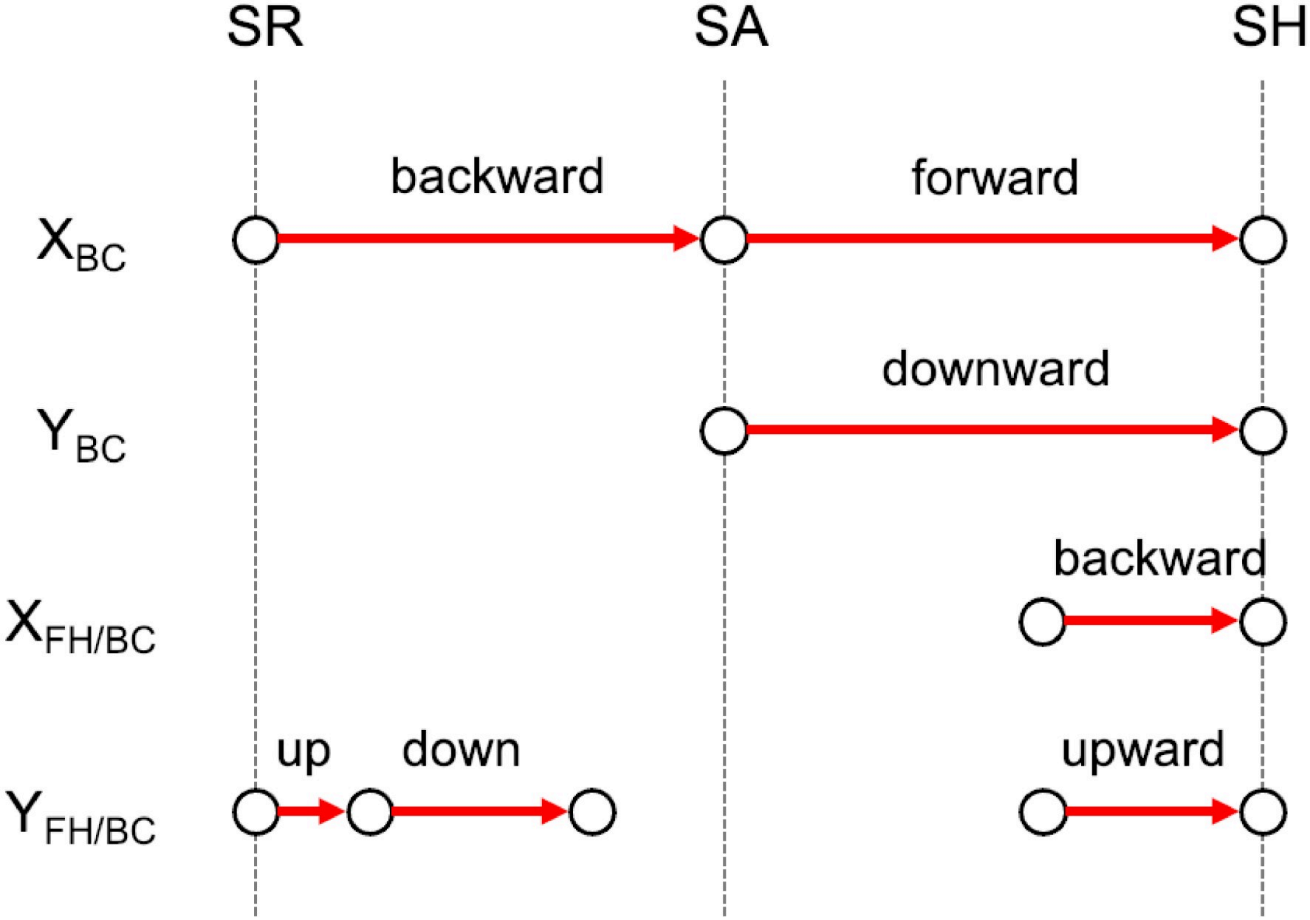
Motion scheme of Steffen’s body center and free hand. The protective gesture during the retreat is represented by arrows for the body center backward and the free hand up/down. The balancing gesture during the lunge attack is represented by arrows for the body center forward/downward and the free hand backward/upward.

Fig 25 shows the motion scheme of Steffen’s body center and free hand. The protective gesture is represented by arrows for the body center backward and the free hand up/down. The balancing gesture is represented by arrows for the body center forward/downward and the free hand backward/upward.

## Applications

### Comparison between lunge and fleche

The lunge and the fleche are two fundamental techniques to attack in fencing. During the lunge, the fencer pushes the front foot forward and extends the back leg. This allows the fencer to maintain his balance after a large step towards the opponent, and it also allows for a return to a defensive position. In contrast, the fleche is an explosive movement generating a surprising hit, the fencer causes an imbalance of the body forward which ends in a crosss-step where the front foot passes behind the back foot.

**Fig 26:**
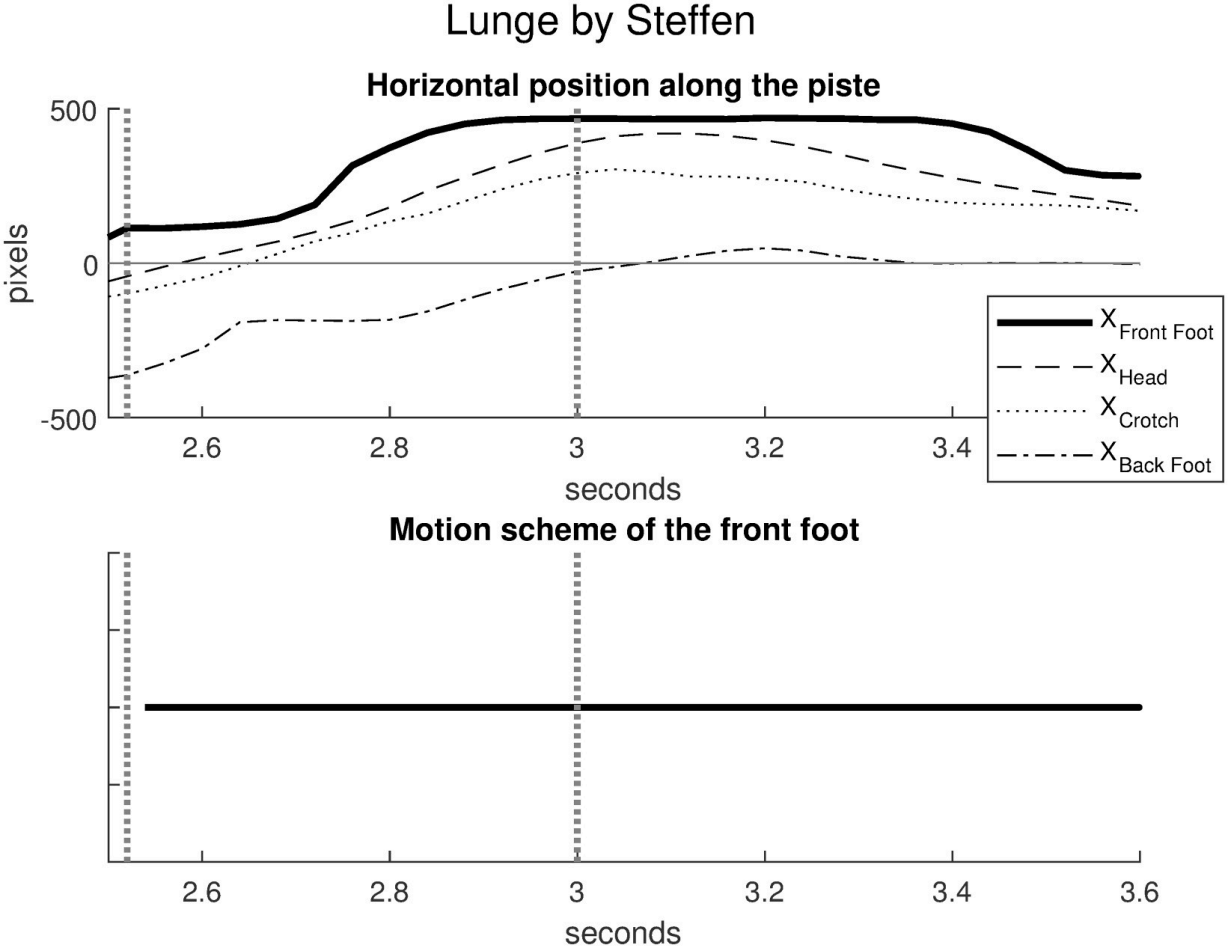
Steffen’s lunge in the duel Steffen-Grumier. The touch at 3 seconds and the start 0.5 seconds before the touch are represented by dotted vertical lines. The motion scheme of the front foot (bottom plot) is monotonic with respect to the head, the crotch and the back foot. Source of the original video: [6].

**Fig 27:**
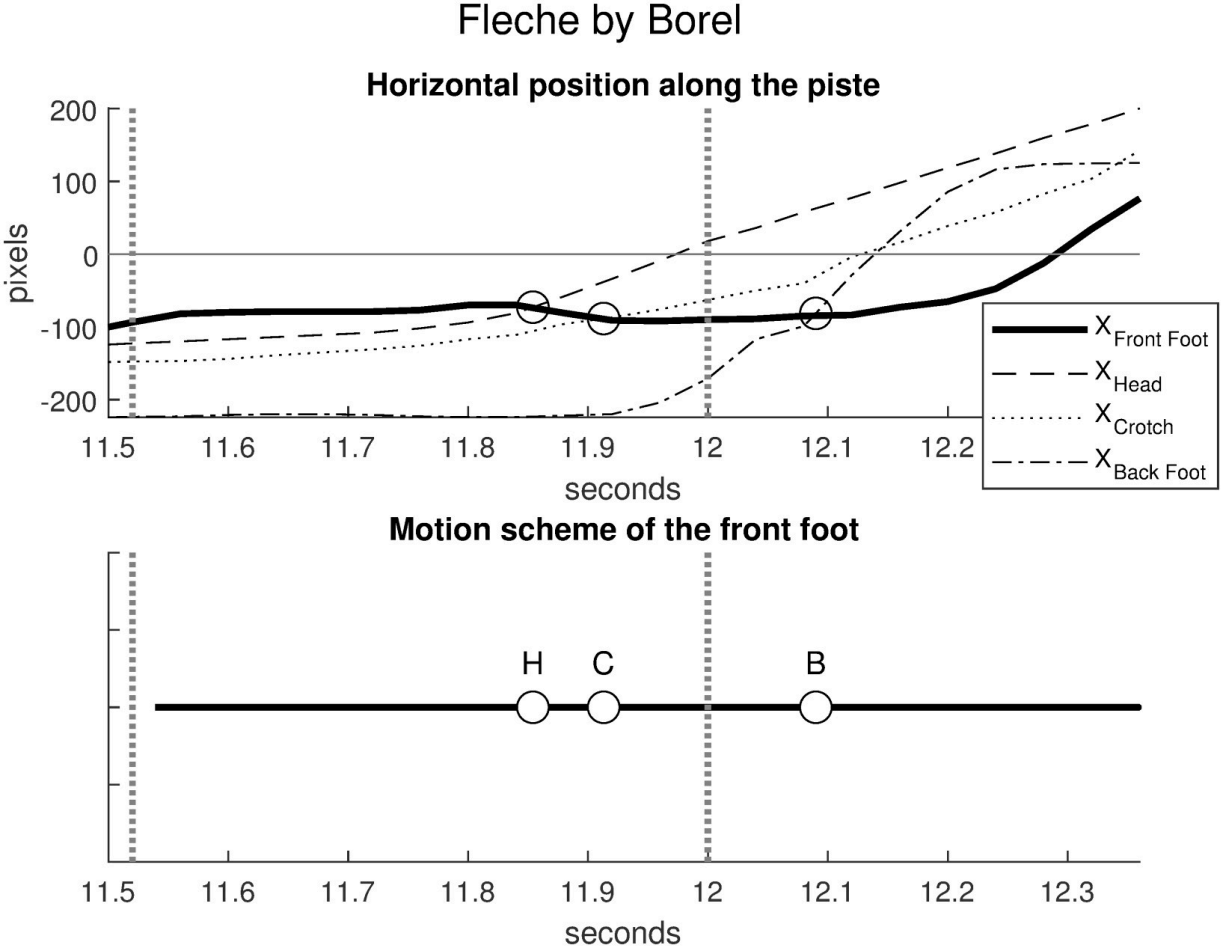
Borel’s fleche in a duel Borel-Svichkar (World Fencing Championships Wuxi, 2018). The touch at 12 seconds and the start 0.5 seconds before the touch are represented by dotted vertical lines. The front foot abscissa remains virtually stable while it is sequentially overtaken by the head (state H), the crotch (state C) and the back foot (state B). The motion scheme of the front foot has three states H, C, B. Source of the original video: [21].

Figs 26 and 27 compare the horizontal positions (in abscissa) of winning attackers in two duels for the front foot, the head, the crotch and the back foot. In both cases, the touch and the start 0.5 seconds before the touch are represented by dotted vertical lines. Fig 26 illustrates the lunge by Steffen in the duel Steffen-Grumier analyzed in this article. Fig 27 illustrates the fleche by Borel, in an official duel Borel-Svichkar [21] (World Fencing Championships, Wuxi 2018, Epee, Semi-final between Yannick Borel, France and Roman Svichkar, Ukraine). Since Steffen is on the left and Borel is on the right, the sign for Borel’s horizontal position has been reversed to make the comparison consistent.

Fig 26 (Steffen’s winning lunge) shows ordered horizontal positions with

$$X_{\text{BackFoot}}<X_{\text{Crotch}}<X_{\text{Head}}<\begin{array}{c} X_{\text{FrontFoot}} \end{array}$$

The motion scheme of the front foot is monotonic with respect to the head, the crotch and the back foot. The front and back feet take forward steps while the crotch and head move steadily forward to the touch, then the feet remain stable after the touch. In accordance with the lunge gesture, this scenario indicates that the fencer propels himself forward while maintaining good balance on the feet.

Fig 27 (Borel’s winning fleche) shows dramatically different movement coordination. The horizontal positions start ordered as with the lunge, but the front foot abscissa remains virtually stable while it is sequentially overtaken by the head (state H), the crotch (state C) and the back foot (state B).

$$\begin{array}{cccccccc} & X_{\text{BackFoot}} & < & X_{\text{Crotch}} & < & X_{\text{Head}} & < & \begin{array}{c} X_{\text{FrontFoot}} \end{array}\\ H⤹\\ & X_{\text{BackFoot}} & < & X_{\text{Crotch}} & < & \begin{array}{c} X_{\text{FrontFoot}} \end{array} & < & X_{\text{Head}}\\ C⤹\\ & X_{\text{BackFoot}} & < & \begin{array}{c} X_{\text{FrontFoot}} \end{array} & < & X_{\text{Crotch}} & < & X_{\text{Head}}\\ B⤹\\ & \begin{array}{c} X_{\text{FrontFoot}} \end{array} & < & X_{\text{BackFoot}} & < & X_{\text{Crotch}} & < & X_{\text{Head}} \end{array}$$

The motion scheme of the front foot has three states H, C, B. Subsequent states are less significant because outside the game rules. In accordance with the fleche gesture, this scenario indicates a fast imbalance of the body forward which ends in a crosss-step.

This comparative measure demonstrates that the concept of motion scheme allows qualitative comparison between two fundamental fencing techniques. It also suggests further quantitative investigations on the fleche gesture using the quantitative tools developed in this article.

### Performance of athletes in a counter-attack

The counter-attack is an offensive action executed against an opponent’s attack. The defender launches his attack after the attacker has launched his, without having first parried or defeated the opponent’s attack.

**Fig 28:**
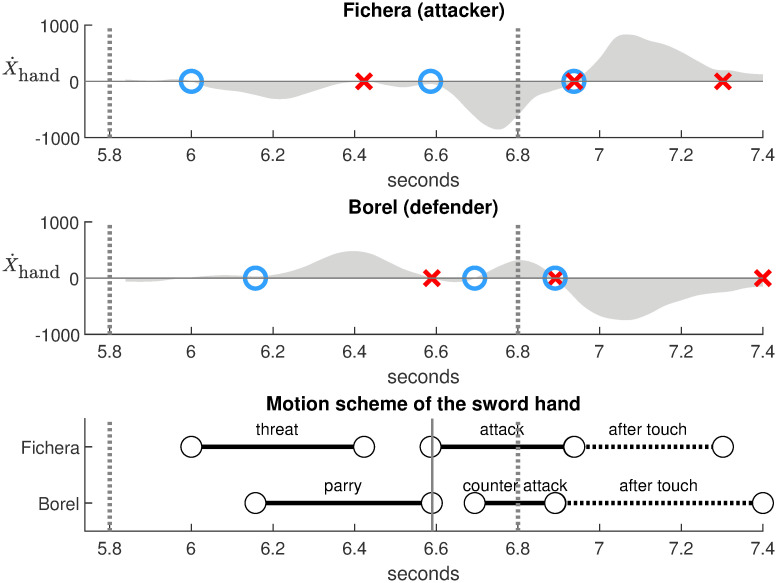
Horizontal velocity of the sword hand during a counter-attack in a duel Borel-Fichera with double touch (Challenge SNCF Réseau, 2017). The touch at 6.8 seconds and the start 1 second before the touch are represented by dotted vertical lines. Each velocity bump (top and middle plots) is delimited between a blue circle and a red cross. The motion scheme (bottom plot) gives subtle indications on the performance of the athletes. It reveals that the attack of Fichera is synchronized with the end of the parry of Borel (continuous vertical line around 6.6 seconds), and the counter-attack of Borel appears more concise than the attack of Fichera. Source of the original video: [22].

Fig 28 shows the analysis of a counter-attack in an official duel Borel-Fichera [22] (Challenge SNCF Réseau, 2017, Epée, Final between Yannick Borel, France and Marco Fichera, Italy). The duel results in a double touch with a direct thrust of Fichera on the forearm and a counter-attack of Borel below.

The top and middle plots in Fig 28 show the horizontal velocity of the sword hand for the attacker Fichera and the defender Borel respectively. The touch at 6.8 seconds and the start 1 second before the touch are represented by dotted vertical lines. The velocity bumps were computed similarly to those of the footwork described earlier in this article. Each velocity bump (gray area) is delimited between a blue circle and a red cross. Velocity is relative to the piste to study the interactive movements of the sword hands between the two fencers.

The velocity bumps can be qualitatively interpreted on the video. Fichera starts with advancing the sword hand to threaten. Borel responds with advancing the sword hand to parry. At the end of the parry, Fichera gives a stronger impulse to attack and touch. Then, Borel gives another impulse to counter-attack and touch.

The bottom plot in Fig 28 summarizes this scenario on a motion scheme determined by the velocity bumps. It is particularly suitable to evaluate the precise timing of the actions giving subtle indications on the performance of the athletes. The continuous vertical line around 6.6 seconds reveals that the attack of Fichera is synchronized with the end of the parry of Borel, which reflects the rapidity of Fichera between decision and action. The counter-attack of Borel appears shorter than the attack of Fichera, which reflects the concision and precision of his gesture.

Thus, this approach provides quantitative indicators on the performance of athletes regarding the timing and synchronization of their movements. This allows a better understanding of fencing actions in space and time, giving details that are not obvious from qualitative video observation.

### New insight on dominance in duels

As explained earlier in this article, the mutual distance between fencers is critical because it reflects how participants constantly adapt their position. It was estimated between the barycenters of the frontal spaces of Steffen and Grumier.

Assuming left and right fencers have horizontal positions *L* and *R* respectively, the mutual distance is given by *D* = *R* − *L*. However, the positions *L* and *R* cannot be deduced from the distance *D* alone.

The center of the duel defined as *C* = (*L* + *R*)/2 is complementary to the distance in the sense that the positions *L* and *R* can be deduced from *D* and *C*, namely *L* = *C* − *D*/2 and *R* = *C* + *D*/2. Thus, the couples of variables (*L*, *R*) and (*D*, *C*) are equivalent.

Like the distance, the center of the duel reflects in another way how participants constantly adapt their position. When the center of the duel moves toward a fencer in one direction, it suggests dominance of the fencer in the opposite direction. For example, if *L* moves to the left and *R* is constant, then *C* moves to the left and dominance of *R* may be interpreted as a retreat of *L*. Similarly, if *L* is constant and *R* moves to the left, then *C* moves to the left and dominance of *R* may be interpreted as an offensive action of *R*.

**Fig 29:**
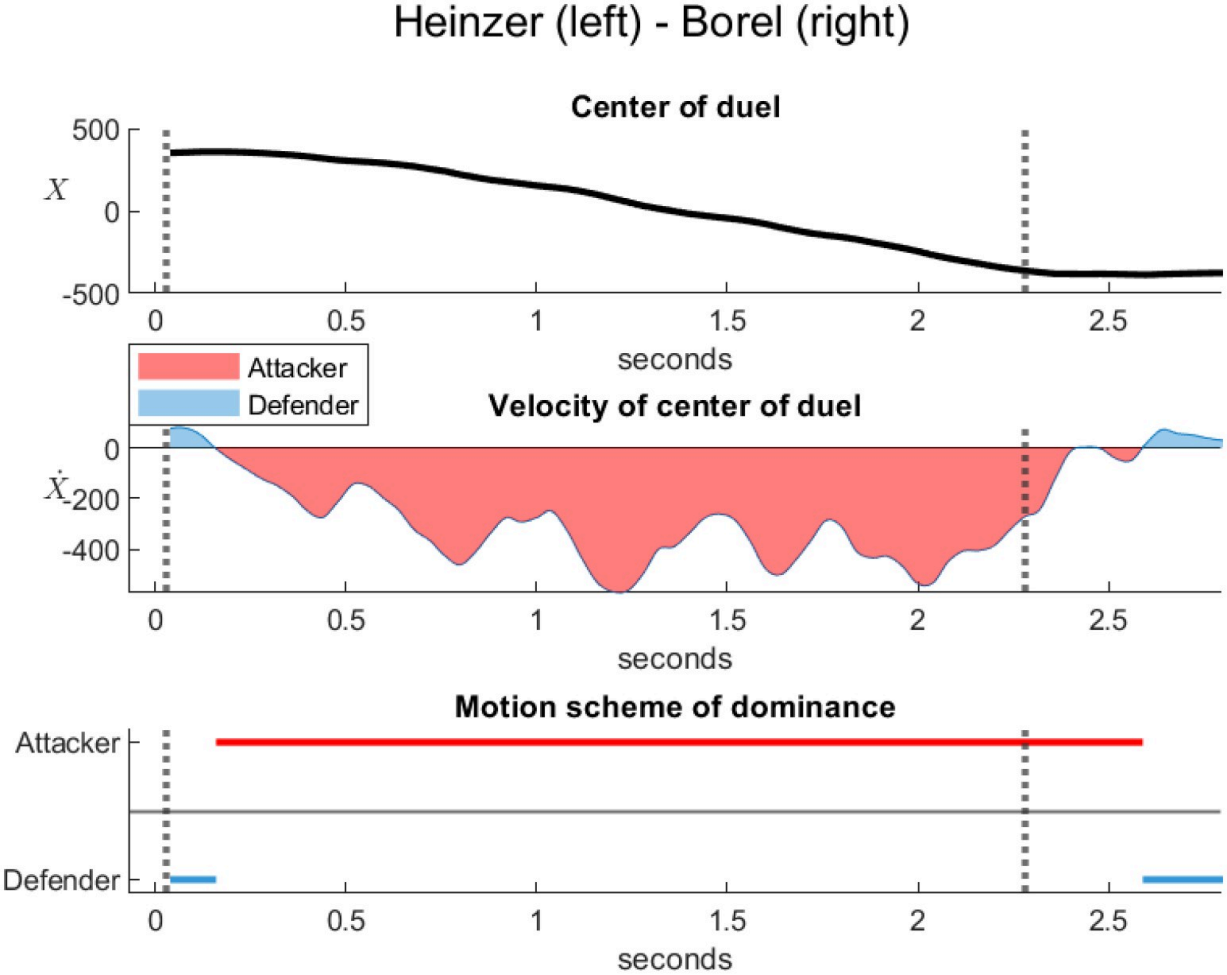
Dominance in a duel between the defender Heinzer and attacker Borel (Championnats d’Europe d’escrime Torun, 2016). The touch at 2.28 seconds and the start 2.25 seconds before the touch are represented by dotted vertical lines. The horizontal position of the center of the duel *X* (top plot) and its velocity $\dot{X}$ (middle plot) were estimated between the barycenters of the frontal spaces. Borel executes a remarkably fluent direct thrust. The motion scheme (bottom plot) indicates the full dominance of the attacker Borel over the defender Heinzer. Source of the original video: [23].

**Fig 30:**
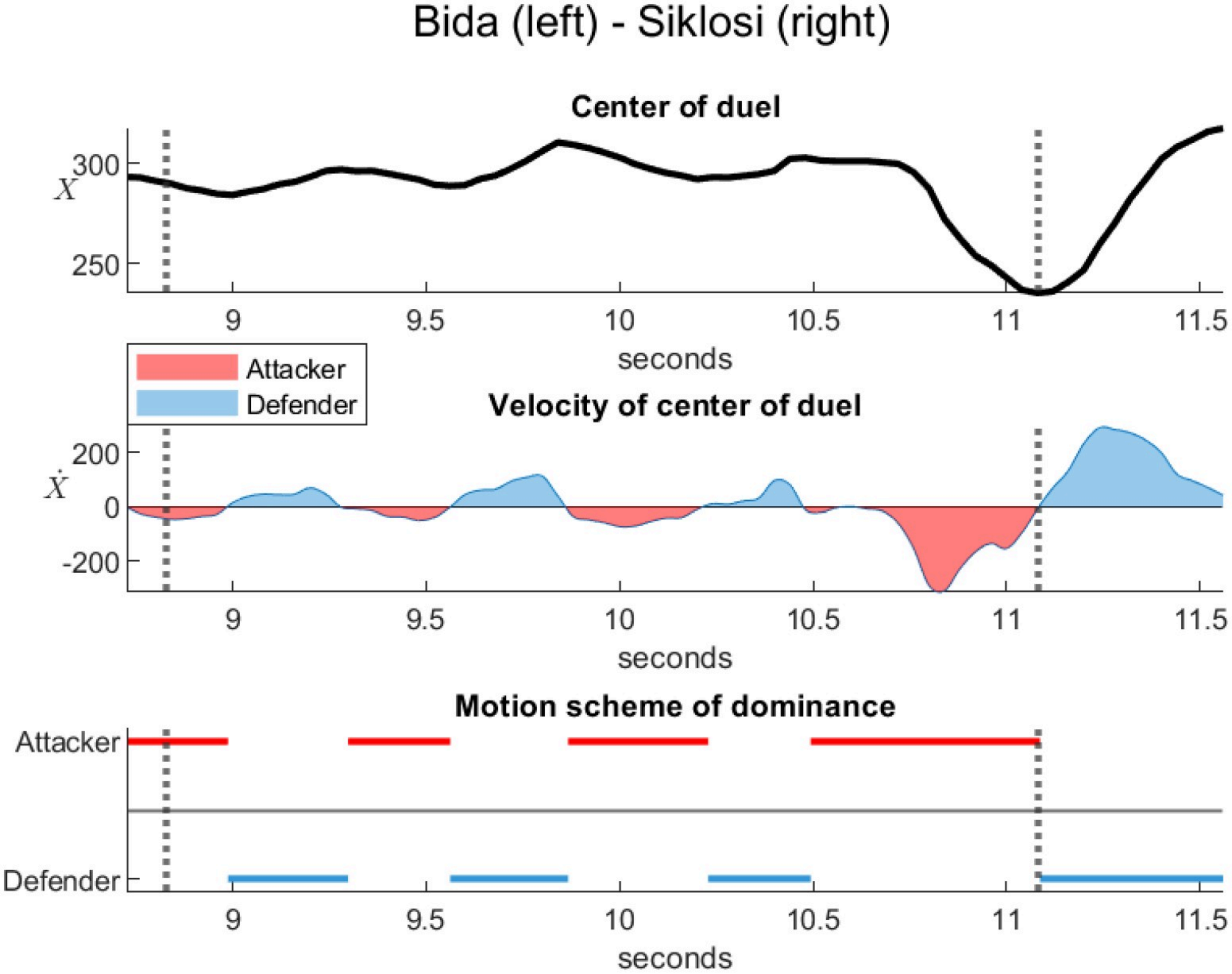
Dominance in a duel between the defender Bida and attacker Siklosi (World Championships Budapest, 2019). The touch at 11.08 seconds and the start 2.25 seconds before the touch are represented by dotted vertical lines. The horizontal position of the center of the duel *X* (top plot) and its velocity $\dot{X}$ (middle plot) were estimated between the barycenters of the frontal spaces. Siklosi executes an explosive direct thrust. The motion scheme (bottom plot) indicates alternating dominance between the defender Bida and the attacker Siklosi, which ends with the touch of Siklosi. Source of the original video: [24].

Figs 29 and 30 analyze the dominance in two official duels during the direct thrust. In both cases, the attacker is on the right and the defender is on the left. The top plots represent the horizontal position *X* of the center of the duel estimated between the barycenters of the frontal spaces. The middle plots represent the velocity $\dot{X}$ of the center of the duel. The red color corresponds to a negative velocity when the center of the duel moves to the left, reflecting the dominance of the right attacker against the left defender. The blue color corresponds to a positive velocity when the center of the duel moves to the right, reflecting the dominance of the left defender against the right attacker. The bottom plots represent the motion scheme of the dominance determined by the sign of the velocity, neglecting small perturbations.

Fig 29 was calculated from an official duel Heinzer-Borel [23] (Championnats d’Europe d’escrime Torun, 2016, Epée, Final between Max Heinzer, Switzerland and Yannick Borel, France). The start and the touch are represented by dotted vertical lines, which correspond to a duration of 2.25 seconds. In this duel, Borel executes a remarkably fluent direct thrust. The opponent is continuously forced to retreat until being hit. The motion scheme indicates the full dominance of the attacker Borel over the defender Heinzer until the touch.

Fig 30 was calculated from an official duel Bida-Siklosi [24] (World Championships Budapest, 2019, Epée, Final between Sergey Bida, Russia and Gergely Siklosi, Hungary). The start and the touch are represented by dotted vertical lines, which correspond to a duration of 2.25 seconds. In this duel, Siklosi executes a beautiful explosive direct thrust. In contrast to Heinzer-Borel, the motion scheme is characterized by alternating dominance between the defender Bida and the attacker Siklosi, which ends with the touch of Siklosi. In addition, the middle plot shows a stronger velocity peak before the touch, consistent with the explosive aspect of the direct thrust.

This study demonstrates that the concept of motion scheme can bring new insight on dominance in duels. It provides a quantitative indicator based on the center of the duel, which is mathematically complementary to the distance between fencers. This dominance reflects the dynamic balance of power between the two fencers, which depends essentially on their tactical-strategic intentions and actions.

### Transferability to other sports: Weightlifting

Due to its generality, the concept of motion scheme can be transferred to sports other than fencing. The method could be applied to Olympic sports filmed in a 2D plane, such as weightlifting [25], climbing [26] or diving [27]. It could also be compared to studies of body movements that can be filmed in a 2D plane, such as yoga postures [28].

In this section, we demonstrate transferability of motion schemes to weightlifting on a video of Lasha Talakhadze (Georgia) [25] who won gold in men’s weightlifting +150kg, in the final at the Rio 2016 Olympic Games. The barbell is lifted in two weightlifting movements called clean and jerk. During the clean, the weightlifter moves the barbell from the floor to shoulder height. During the jerk, the weightlifter lifts the barbell above his head, then holds it with his arms and legs straight. Since this article focuses on fencing, the purpose of this section is not to make an in-depth study of weightlifting but essentially to show how the method can be transferred to a discipline radically different from fencing.

**Fig 31:**
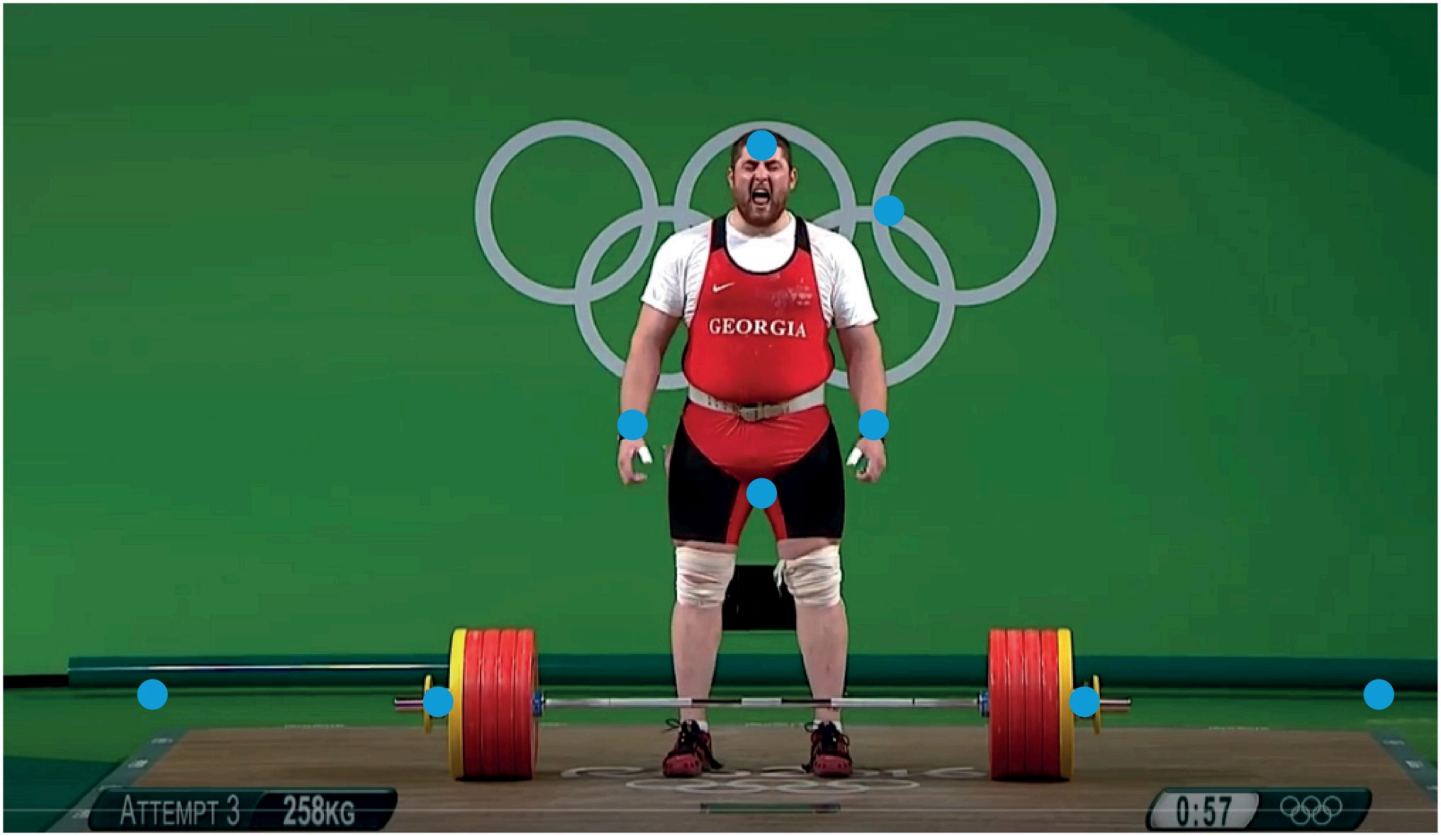
Lasha Talakhadze in men’s weightlifting. The video tracking was based on 9 points of interest represented by blue markers, including 3 fixed points of calibration, 2 points on the barbell and 4 anatomical points (forehead, crotch, wrist on the left, wrist on the right). Source of the original video: [25].

Fig 31 shows an image capture with 9 points of interest represented by blue markers, including 3 fixed points of calibration, 2 points on the barbell and 4 anatomical points (forehead, crotch, wrist on the left, wrist on the right).

**Fig 32:**
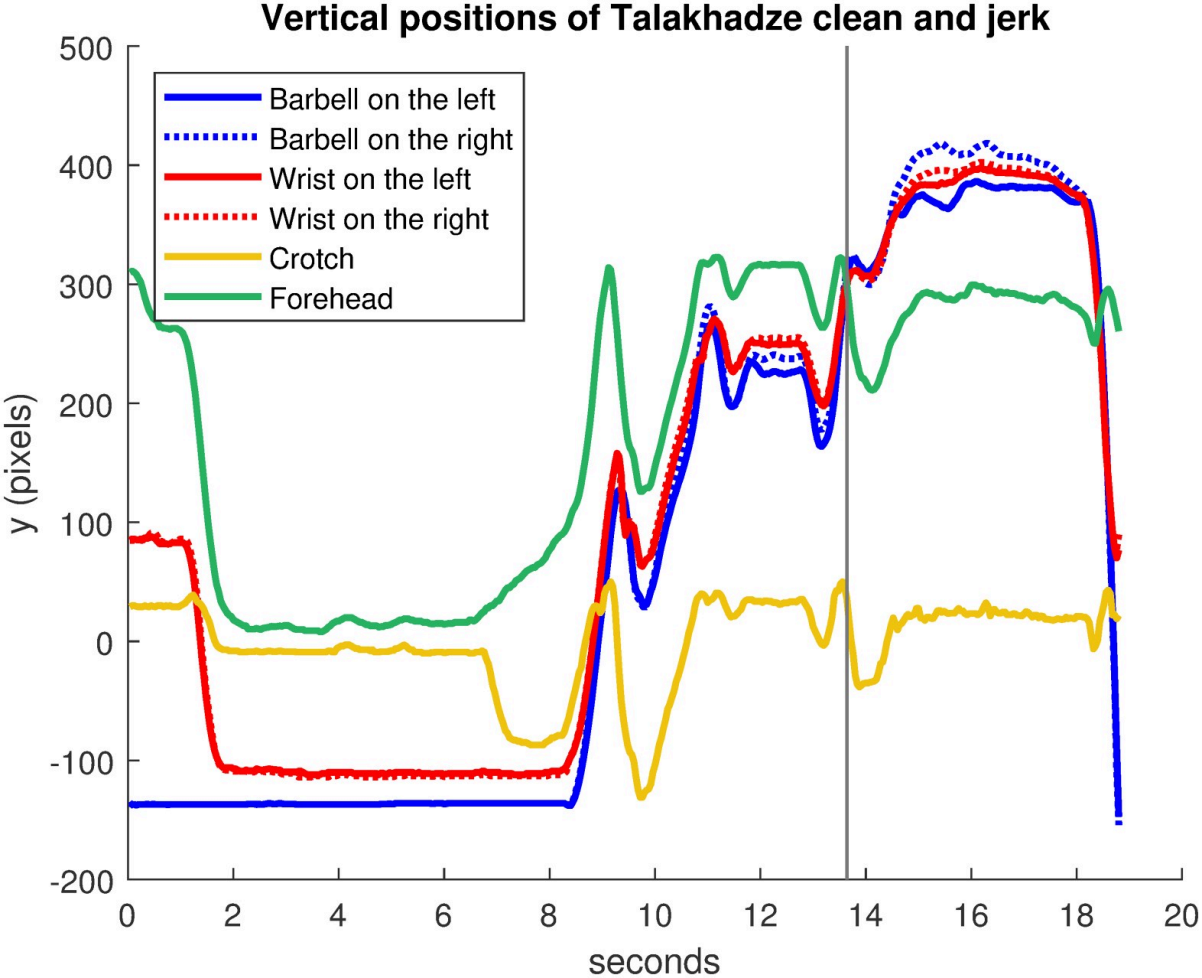
Vertical positions of Talakhadze clean and jerk. Curves of vertical positions (ordinate) of points of interest for Talakhadze and the barbell. The black vertical line at 13.64 seconds indicates when the barbell passes above the forehead.

Fig 32 shows the curves of vertical positions (ordinate) of points of interest for Talakhadze and the barbell captured by video tracking. Informally, the barbell (blue curves) is lifted from the ground upwards. The wrists (red curves) follow the movement of the barbell they control. The black vertical line at 13.64 seconds corresponds to the critical moment when the barbell (blue curves) passes above the forehead (green curve). At the end, the barbell is quickly released as the wrists descend.

**Fig 33:**
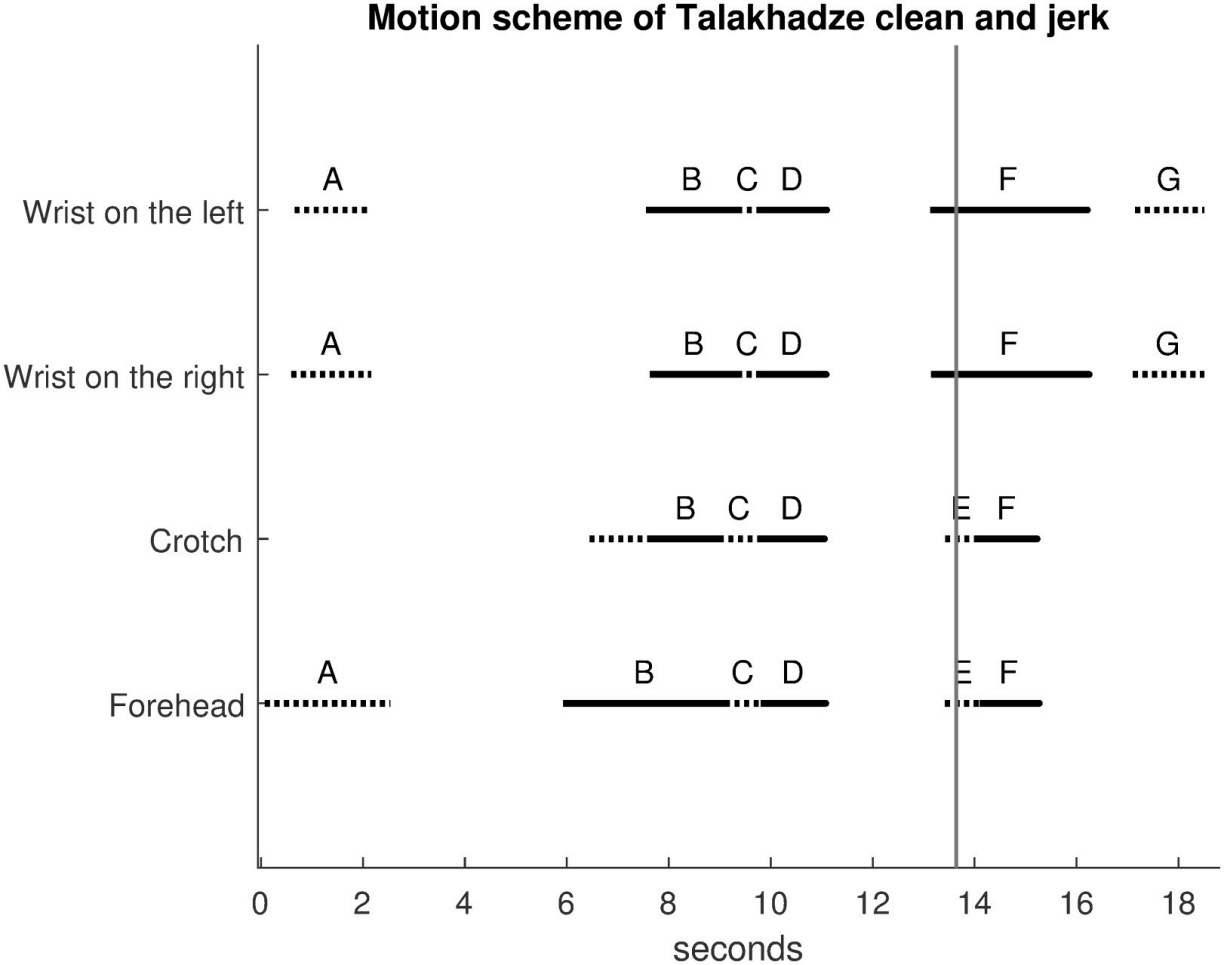
Motion scheme of Talakhadze clean and jerk. The motion scheme for Talakhadze describes precise timing of movements. The continuous segments correspond to upward movements while the dotted segments correspond to downward movements. The black vertical line at 13.64 seconds indicates when the barbell passes over the forehead.

Fig 33 shows the motion scheme for Talakhadze with precise timing description of movements. It was calculated using the same velocity bump method as described earlier in this article, neglecting small velocity peaks to simplify the diagram. Velocity curves were derived from the trajectories in Fig 32, but they are not shown for conciseness. The continuous segments correspond to upward movements while the dotted segments correspond to downward movements.

The motion scheme on Fig 33 is numbered with letters A, B, C, D, E, F, G corresponding to the following movements of Talakhadze in accordance with curves on Fig 32 extracted from the video:

- A: He takes the bar on the ground
- B: He rises up
- C: He falls quickly and recovers the bar in a crouched position
- D: He rises up; the “clean” phase is done
- E: He lowers again to be able to grasp the barbell with arms extended above the head; it is the “jerk” phase
- F: He rises up to reach the final position
- G: He drops the bar by lowering his hands

Therefore, motion schemes can be used to analyze movements in sports other than fencing. Their formal aspect is important from a scientific point of view to extract quantitative indicators, as well as to facilitate qualitative interpretations. Intuitively, motion schemes provide a kind of language that reduces sports exercices to movements phrases.

## Discussion

### Scientific value

The science of fencing is more than an art for Agrippa, who was a mathematician, architect, and engineer. He was importantly influenced by the science of his time, including Euclidean geometry and Aristotelian physics. His Treatise on the Science of Arms prefigures modern calculations of geometrical shapes in space and time applied to fencing gestures. As such, it is not just the treatise of a fencing master describing the art of effective techniques. He made a significant effort to scientifically analyze this art, extract fundamental principles and give empirical proofs. From an intellectual point of view, Agrippa published his treatise just before the birth of Galileo Galilei at the beginning of modern science.

The concept of motion scheme revisits the scientific conception of Agrippa by using modern mathematical and computational techniques. While Agrippa developed his geometrical approach based on approximate observations of duels, motions schemes have been developed to make precise quantitative measurements applicable to an industrial amount of fencing duel videos available on YouTube and other websites. In this article, the concept of motion scheme is illustrated on a unique example between Steffen and Grumier, which was carefully chosen by fencing experts to be a representative direct thrust with lunge. Such unique analysis is essential to better understand fencing actions and judge the performance of two athletes in an official duel. In contrast, statistical analyzes give averages but no precise information about the outcome of a particular duel.

The scientific value of this article is fundamentally anchored in sports research. Video analysis methods have long been used to study many sports. In a review of vision-based motion analysis in sport, Barris and Button [29] discuss how data recording systems are useful to measure human physical activity. Sport scientists calculate player activity with their constraints, in which velocities and accelerations reveal performance profiles to describe movement patterns efficiently. Thus, the approach to geometrical fencing through motion schemes fits perfectly into the framework of sports science.

From an experimental point of view, motion schemes have the advantage of being non-invasive. The scientific method does not disturb the fencers with sensors and does not require the introduction of a specific device on the ground.

Another scientific interest of this article is that the approach is sportingly realistic. Indeed, in official competitions, fencing duels are practiced on a real ground between two human opponents with constraints on the piste and strict international rules imposed on their actions. Furthermore, Olympic medals are won in a context of coaching, preparation, events, health, injuries, psychological pressure and many other elements difficult or impossible to reproduce in laboratory experiments.

The main originality of this article lies in the formalization of a fencing duel as a directed graph in which each arrow describes the movement of a dynamic region of interest. This analytical approach breaks down a duel into more elementary spatial elements (motion spaces). It greatly improves the scientific interpretation of the art of fencing with diagrams of the precise timing of movements. It allows quantitative comparison with the literature, such as the knee and ankle rotations, or the lunge. It provides quantitative features on coordination patterns, such as the correlation matrix computed from a kinematic model. It can reveal subtle interactive behaviors, such as protective movement in response to a threat. It can reveal tactical-strategic choices of fencers, such as footwork reflecting forward and backward steps, or the decision to attack after a parry.

### Principle of uniqueness

This article has introduced motion schemes to study the unique characteristics of duels. It is not a statistical method for studying cohorts of fencers in the laboratory but rather a geometrical method producing personalized portraits of fencers in real field conditions. There are at least three fundamental reasons to consider the unique characteristics of a duel as presented in this article.

As Chen et al. [17] pointed out, fencing is an idiosyncratic sport involving unique movement patterns. Each duel has a unique story. For instance, Steffen’s direct thrust is not a stereotypical gesture but a personally crafted three-step walk to score the touch. The three decisive steps in the attack are facilitated by an increase in distance after the mutual retreat of the two players, which is itself a consequence of Grumier’s threat. Thus, each duel determines a chain of actions according to the rules and contingencies of the game. The dynamic choices made by the players reflect their creativity. As in chess, the face to face between fencers is fundamentally collaborative but also highly strategic because they keep their plans and tricks secret.

The uniqueness of combat situations is addressed by Master Sicard [30]. An action never occurs twice in the same way. The duel evolves contingently. The subjects must permanently adapt their game to rhythms, distances and environment. Master Sicard also describes the uniqueness of individuals. Each individual is unique in style, body shape and personality. This has to be taken into account in training to know the strengths and weaknesses during competitions. The fighting style is influenced by the country (German, Italian, …), physical skills, register of techniques, as well as individual strategic qualities of fencers. In addition, the types of movements depend on the specialization of the fencers. Indeed, Dedieu et al. [31] reveal significant differences in kinematics and coordination between foil and épée during the execution of the lunge. Motion schemes could be used in future work to investigate the concept of fighting style from videos of training and official competitions.

The previous sections have demonstrated the use of motion schemes to decompose complex chains of actions into fundamental movements (footsteps, knee flexion/extension, …) The complexity of fencing gestures tends to produce unique expressions making it difficult for the opponent to predict the action. This is consistent with the findings of Mantovani et al. [32] who developed an algorithm to recognize the movements of elite fencing athletes under controlled conditions. For constrained movements, recognition was efficient in most cases. However, for free movements similar to those in real competitions, athletes not only performed fundamental movements but also combined different movements to surprise the opponent.

### Movement performance

Each transition in a motion scheme represents a movement with a bodily realization in physical space. The previous sections have shown the importance of shape and velocity of fencing movements, which are controlled to optimize motor tasks for athletic performance. Interestingly, Flash et al. [33] suggest that optimization principles can produce relationships between geometrical form and velocity concerning human arm movements. Similar works have been done on the isochrony principle by Viviani et al. [34], the 2/3 power law by Lacquaniti et al. [35] and its extension to human locomotion by Hicheur et al. [36].

Fencing duels take place in highly dynamic and unpredictable environments reminiscent of Poulton’s definition of open skills [37]. Fencers have their own style and personality to deal with different situations in duels. Master Sicard [11] discriminates between three fencing styles, which are physical (based on force, speed), academic (based on a technical catalogue) and strategic (based on reflection, tactical-strategic qualities).

Precise execution of movements depends very much on motor skills acquired through training and experience. In 1975, Schmidt [38] proposed that motor skills could be memorized in schemas. A schema is a general motor program describing a class of similar movements. This approach takes into account the variability of movements, which means that a movement performed several times is never exactly the same, in accordance with the principle of uniqueness discussed previously. Such variability is induced by the abundance of degrees of freedom as described by Bernstein [39].

### Spatial representation

Fencing actions determine units of space in which they take place. Each transition in a motion scheme represents the time evolution of a unit of space defined as a spatial relationship between points of interest in the scene. As pointed out by Berthoz [40], the brain constructs units of space related to action, which suggests looking for connections with the neurophysiological foundations of spatial representation.

The modular representation of space in the brain is discussed by Clery et al. [41] and Bennequin et al. [42]. Some cortical areas represent the peripersonal space (or near space), which is the space surrounding the subject and that can be acted by the body. Other cortical regions represent the extrapersonal space (or far space), which cannot be directly acted by the body but can be reached with short locomotion. These cortical areas have been investigated experimentally in clinical studies [43].

As reported by Clery et al. [41], the peripersonal space representation involves a parieto-premotor neuronal network with bimodal visuo-tactile neurons responding both to tactile stimuli and visual stimuli in the near space. More precisely, the near space around the head involves the parietal area VIP while the near space around the arm/hand involves the premotor area F4. This leads to refining the concept of peripersonal space into multiple peripersonal spaces, which are distinct but functionnally coupled.

The boundary of peripersonal space is not fixed. Berti et al. [44] have studied how the peripersonal space can be extended when the subject manipulates a tool. According to this idea, the sword handled by a fencer may extend the peripersonal space during a fight. Furthermore, Brozzoli et al. [45] have investigated a functional link between multisensory peripersonal space and voluntary actions. Perhaps the peripersonal space depends on the action in a fencing duel.

As proposed by Bennequin [46], the notion of point and trajectory to be controlled and adapted could be replaced by a notion of transformation between spaces. There would be a space of positions and moves in the physical world, as well as a space of postures and paths between postures. Similarly, the states and transitions of a motion scheme can be interpreted as postures and paths between postures, the directed graph of which is constructed on the fly by the fencers during a duel.

## References

[1] Agrippa C. Trattato di scientia d’arme: con vn dialogo di filosofia. Facsimile Publisher; 2018.

[2] Mondschein K. The Number of Motion: Camillo Agrippa’s Geometrical Fencing and the Enumeration of the Body. Journal of the Northern Renaissance. 2014;(6).

[3] Green TA, Svinth JR, editors. Martial arts of the world: an encyclopedia of history and innovation. Santa Barbara, Calif: ABC-CLIO; 2010.

[4] Cesariano C. Di Lucio Vitruuio Pollione de Architectura Libri Dece; 1521.

[5] Fowler CO. From a Geometry of Vision to a Geometry of Light in Early-Modern Perspective. Architecture_MPS. 2017. doi: 10.14324/111.444.amps.2017v11i1.001

[6] Olympic games 2016. Steffen-Grumier duel. Men’s Epee Bronze Medal Bout. Sequence extracted from 00:01:48:14 to 00:01:52:05 (hh:mm:ss:number at 25 images/s); 2016. Available from: https://www.youtube.com/watch?v=lR3BX9OBcDs.

[7] Bauer M, Bruveris M, Michor PW. Overview of the Geometries of Shape Spaces and Diffeomorphism Groups. Journal of Mathematical Imaging and Vision. 2014;50(1-2):60–97. doi: 10.1007/s10851-013-0490-z

[8] Poincaré H. La Science Et l’Hypothèse. Wentworth Press; 2018.

[9] Hatcher A. Algebraic topology. Cambridge; New York: Cambridge University Press; 2002.

[10] Qu B, Kumar P, Zhang E, Jaiswal P, Cooper L, Elser J, et al. Interactive design and visualization of N-ary relationships. In: SIGGRAPH Asia 2017 Symposium on Visualization on—SA’17. Bangkok, Thailand: ACM Press; 2017. p. 1–8. Available from: http://dl.acm.org/citation.cfm?doid=3139295.3139314.

[11] Sicard M. S’entraîner à sentir en épée. Les Cahiers de l’Entraîneur; 2007.

[12] Trautmann C, Martinelli N, Rosenbaum D. Foot loading characteristics during three fencing-specific movements. Journal of Sports Sciences. 2011;29(15):1585–1592. doi: 10.1080/02640414.2011.605458 22077403

[13] Andriacchi TP, Alexander EJ. Studies of human locomotion: past, present and future. Journal of Biomechanics. 2000;33(10):1217–1224. doi: 10.1016/S0021-9290(00)00061-0 10899330

[14] Faisal AI, Majumder S, Mondal T, Cowan D, Naseh S, Deen MJ. Monitoring Methods of Human Body Joints: State-of-the-Art and Research Challenges. Sensors. 2019;19(11):2629. doi: 10.3390/s19112629 31185629 PMC6603670

[15] Mentiplay BF, Banky M, Clark RA, Kahn MB, Williams G. Lower limb angular velocity during walking at various speeds. Gait & Posture. 2018;65:190–196. doi: 10.1016/j.gaitpost.2018.06.162 30558929

[16] Jessop DM, Pain MTG. Maximum Velocities in Flexion and Extension Actions for Sport. Journal of Human Kinetics. 2016;50(1):37–44. doi: 10.1515/hukin-2015-0139 28149339 PMC5260637

[17] Chen TLW, Wong DWC, Wang Y, Ren S, Yan F, Zhang M. Biomechanics of fencing sport: A scoping review. PLOS ONE. 2017;12(2):e0171578. doi: 10.1371/journal.pone.0171578 28187164 PMC5302478

[18] Gutierrez-Davila M, Rojas FJ, Antonio R, Navarro E. Response timing in the lunge and target change in elite versus medium-level fencers. European Journal of Sport Science. 2013;13(4):364–371. doi: 10.1080/17461391.2011.635704 23834541

[19] Netter FH. Atlas of human anatomy. Philadeplhia: Elsevier; 2019.

[20] Chatauvillard LALB. Essai Sur Le Duel. Paris, chez Bohaire; 1836.

[21] World Fencing Championships Wuxi 2018. Borel-Svichkar duel. Men’s Epee Semi-final. Sequence extracted from 00:09:06:24 to 00:09:19:07 (hh:mm:ss:number at 25 images/s); 2018. Available from: https://www.youtube.com/watch?v=tBZ4JEmxcII.

[22] Challenge SNCF Réseau 2017. Borel-Fichera duel. Men’s Epee Final. Sequence extracted from 00:05:30:00 to 00:05:37:09 (hh:mm:ss:number at 25 images/s); 2017. Available from: https://www.youtube.com/watch?v=1CS66m1Y404.

[23] Championnats d’Europe d’escrime Torun 2016. Heinzer-Borel duel. Men’s Epee Final. Sequence extracted from 00:04:37:07 to 00:04:40:02 (hh:mm:ss:number at 25 images/s); 2016. Available from: https://www.youtube.com/watch?v=odAqXAmRsgA.

[24] World Championships Budapest 2019. Bida-Siklosi duel. Men’s Epee Final. Sequence extracted from 01:44:38:18 to 01:44:50:06 (hh:mm:ss:number at 25 images/s); 2019. Available from: https://www.youtube.com/watch?v=vYN_Gz1ZpAc.

[25] Olympic games 2016. Rio Replay: Men’s +105kg Weightlifting Final. Sequence extracted from 00:03:42:22 to 00:04:01:16 (hh:mm:ss:number at 25 images/s); 2016. Available from: https://www.youtube.com/watch?v=EiqEFUFM-KI.

[26] International Federation of Sport Climbing. IFSC World Cup Chongqing 2019 | Speed finals. Example of exercise filmed in a 2D plane at 49:05 (mm:ss); 2019. Available from: https://www.youtube.com/watch?v=lBf2lp7OxnY.

[27] Olympic Games 2012. Diving—Women’s 10m Platform—Final | London 2012 Olympic Games. Example of exercise filmed in a 2D plane at 19:15 (mm:ss); 2012. Available from: https://www.youtube.com/watch?v=QhXToslnPvA.

[28] Wu Z, Zhang J, Chen K, Fu C. Yoga Posture Recognition and Quantitative Evaluation with Wearable Sensors Based on Two-Stage Classifier and Prior Bayesian Network. Sensors. 2019;19(23):5129. doi: 10.3390/s19235129 31771131 PMC6929085

[29] Barris S, Button C. A Review of Vision-Based Motion Analysis in Sport. Sports Medicine. 2008;38(12):1025–1043. doi: 10.2165/00007256-200838120-00006 19026019

[30] Sicard M. Model for an epée combat. Calaméo; 2015.

[31] Dedieu P, Champain G, Salesse M, Zanone PG. Influence de la spécialisation sur la réalisation de la fente en escrime. Calaméo; 2015.

[32] Mantovani G, Ravaschio A, Piaggi P, Landi A. Fine classification of complex motion pattern in fencing. Procedia Engineering. 2010;2(2):3423–3428. doi: 10.1016/j.proeng.2010.04.168

[33] Flash T, Handzel AA. Affine differential geometry analysis of human arm movements. Biological Cybernetics. 2007;96(6):577–601. doi: 10.1007/s00422-007-0145-5 17406889 PMC2799626

[34] Viviani P, Terzuolo C. Trajectory determines movement dynamics. Neuroscience. 1982;7(2):431–437. doi: 10.1016/0306-4522(82)90277-9 7078732

[35] Lacquaniti F, Terzuolo C, Viviani P. The law relating the kinematic and figural aspects of drawing movements. Acta Psychologica. 1983;54(1-3):115–130. doi: 10.1016/0001-6918(83)90027-6 6666647

[36] Hicheur H, Vieilledent S, Richardson MJE, Flash T, Berthoz A. Velocity and curvature in human locomotion along complex curved paths: a comparison with hand movements. Experimental Brain Research. 2005;162(2):145–154. doi: 10.1007/s00221-004-2122-8 15586276

[37] Poulton EC. On prediction in skilled movements. Psychological Bulletin. 1957;54(6):467–478. doi: 10.1037/h0045515 13485273

[38] Schmidt RA. A schema theory of discrete motor skill learning. Psychological Review. 1975;82(4):225–260. doi: 10.1037/h0076770

[39] Bernstein N. The Coordination and Regulation of Movements. Pergamon Press; 1967.

[40] Berthoz A. The brain’s sense of movement. Cambridge, Mass: Harvard University Press; 2000.

[41] Cléry J, Guipponi O, Wardak C, Ben Hamed S. Neuronal bases of peripersonal and extrapersonal spaces, their plasticity and their dynamics: Knowns and unknowns. Neuropsychologia. 2015;70:313–326. doi: 10.1016/j.neuropsychologia.2014.10.022 25447371

[42] Bennequin D, Berthoz A. Several Geometries for Movements Generations. In: Geometric and Numerical Foundations of Movements. vol. 117. Cham: Springer International Publishing; 2017. p. 13–42. Available from: http://link.springer.com/10.1007/978-3-319-51547-2_2.

[43] Weiss PH, Marshall JC, Wunderlich G, Tellmann L, Halligan PW, Freund HJ, et al. Neural consequences of acting in near versus far space: a physiological basis for clinical dissociations. Brain: A Journal of Neurology. 2000;123 Pt 12:2531–2541. doi: 10.1093/brain/123.12.2531 11099454

[44] Berti A, Frassinetti F. When far becomes near: remapping of space by tool use. Journal of Cognitive Neuroscience. 2000;12(3):415–420. doi: 10.1162/089892900562237 10931768

[45] Brozzoli C, Cardinali L, Pavani F, Farnè A. Action-specific remapping of peripersonal space. Neuropsychologia. 2010;48(3):796–802. doi: 10.1016/j.neuropsychologia.2009.10.009 19837102

[46] Bennequin D. Les chemins et les espaces de la géométrie, de Babylone à Bures-sur-Yvette. In: Les arts de la mémoire et les images mentales. Collège de France; 2018. p. 165–175. Available from: http://books.openedition.org/cdf/5559.

